# A systematic benchmark of bioinformatics methods for single-cell and spatial RNA-seq nanopore long reads data

**DOI:** 10.1093/nargab/lqag070

**Published:** 2026-07-06

**Authors:** Ali Hamraoui, Audrey Onfroy, Catherine Sénamaud-Beaufort, Fanny Coulpier, Sophie Lemoine, Laurent Jourdren, Morgane Thomas-Chollier

**Affiliations:** GenomiqueENS, Institut de Biologie de l’ENS (IBENS), Département de biologie, École normale supérieure, CNRS, INSERM, Université PSL, 75005 Paris, France; Group Bacterial Infection, Response & Dynamics, Institut de biologie de l’ENS (IBENS), École normale supérieure, CNRS, INSERM, Université PSL, 75005 Paris, France; Team Neurofibromatosis and Lymphoma Oncogenesis, Institut Mondor de Recherche Biomédicale, UPEC, INSERM, 94000 Créteil, France; GenomiqueENS, Institut de Biologie de l’ENS (IBENS), Département de biologie, École normale supérieure, CNRS, INSERM, Université PSL, 75005 Paris, France; Team Neurofibromatosis and Lymphoma Oncogenesis, Institut Mondor de Recherche Biomédicale, UPEC, INSERM, 94000 Créteil, France; GenomiqueENS, Institut de Biologie de l’ENS (IBENS), Département de biologie, École normale supérieure, CNRS, INSERM, Université PSL, 75005 Paris, France; GenomiqueENS, Institut de Biologie de l’ENS (IBENS), Département de biologie, École normale supérieure, CNRS, INSERM, Université PSL, 75005 Paris, France; GenomiqueENS, Institut de Biologie de l’ENS (IBENS), Département de biologie, École normale supérieure, CNRS, INSERM, Université PSL, 75005 Paris, France; Group Bacterial Infection, Response & Dynamics, Institut de biologie de l’ENS (IBENS), École normale supérieure, CNRS, INSERM, Université PSL, 75005 Paris, France

## Abstract

Alternative splicing plays a crucial role in transcriptomic complexity, yet remains difficult to resolve at the single-cell level due to the limitations of short-read technologies. Coupling single-cell with long-read sequencing offers full-length transcript coverage, enabling more accurate isoform detection. Diverse computational tools tailored for single-cell and spatial long-read transcriptomics have been developed. To compare the effectiveness of these approaches, we generated paired short-read and Nanopore long-read single-cell datasets, tailored for benchmarking bioinformatics tools. We evaluated ten state-of-the-art methods, spanning four analytical dimensions: barcodes and unique molecular identifiers (UMI) detection, demultiplexing and UMI clustering, gene-level expression profiling, and isoform detection and quantification. Using real and simulated datasets across different protocols, sequencing depths and chemistries, we assessed the accuracy, robustness, and scalability of each tool. Our results revealed method-specific trade-offs, and highlight the importance of sequencing quality and UMI correction strategies. This benchmark provides a practical resource for optimizing isoform analysis and accurate gene expression profiling in single-cell and spatial transcriptomics using long-read sequencing. The workflow employed for benchmarking is designed to be reusable, thereby enabling method developers to compare their own approaches against the set of reference methods evaluated in this work.

## Graphical Abstract

**Graphical Abstract:**
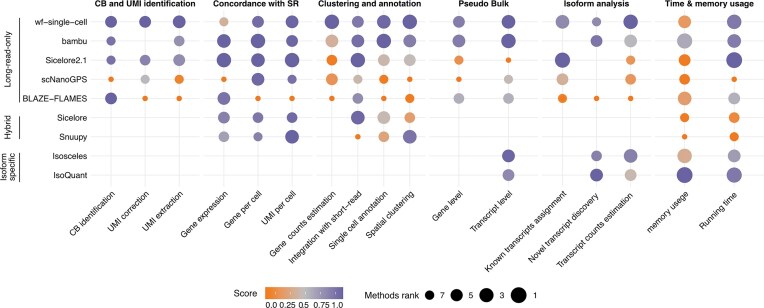

## Introduction

Alternative splicing (AS) significantly contributes to transcriptome complexity and has critical implications for cellular functions. While identifying isoforms can be achieved using bulk RNA sequencing (RNA-seq), this approach relies on RNA pooled from thousands of cells, resulting in averaged transcriptome information that ignores cell heterogeneity [1]. Recent advancements in single-cell isolation and capture techniques, such as droplet-based methods [2, 3], and *in situ*-capture-based methods [4, 5] have enabled high-throughput quantification of gene expression at both single-cell resolution and in spatially resolved contexts.

However, high-throughput full-length RNA isoform identification and quantification remain challenging at the single-cell level. Typical droplet-based single-cell protocols, sequenced in short reads, capture only limited sequence information. This information is typically restricted to one end of the transcript (e.g. 10X Genomics 3′ Chemistry) and does not span exon-exon junctions across full-length isoforms. As a result, the comprehensive detection and quantification of alternative isoforms remains challenging [6, 7].

Plate-based protocols (e.g. smart-seq) are used as an alternative, for their high sensitivity and ability to capture entire transcripts [8]. Nevertheless, they still suffer from intrinsic limitations of short-read technologies, leading to isoform reconstruction errors caused by ambiguous read-to-isoform mapping and 5′ coverage bias [9, 10]. Furthermore, their limited throughput—generally around 400 cells per experiment—and relatively high cost per cell, reduce their suitability for large-scale studies [11, 12].

Long-read sequencing technologies, including Oxford Nanopore Technology (ONT) and Pacific Biosciences (PacBio), overcome these limitations by enabling the unambiguous identification of complete exon structures [13]. These sequencing platforms were previously limited by high sequencing error rates and/or low throughput. For single-cell approaches, this hampered the accurate detection of barcodes and unique molecular identifiers (UMIs), thus limiting the representation of the whole diversity of captured RNA transcripts [14]. However, improvements of platforms and library preparation methods over the past five years have enhanced accuracy and throughput, with tens of millions of reads generated in a single experiment [15].

Beside technological development, several computational methods have been developed to specifically address bioinformatics challenges associated with processing long-read single-cell RNA sequencing (scRNA-seq), including spatial data. A comprehensive overview of these methodological developments was recently provided by [16] and [17]. Tools such as Sicelore [18], Snuupy [19], ScNapBar [20], and scTagger [21] focus on barcodes and UMI assignments by integrating paired short-read data. In contrast, approaches like Sicelore 2.1 [18], wf-single-cell (https://github.com/epi2me-labs/wf-single-cell), scNanoGPS [22], Longcell [23], and Scywalker [24] perform barcodes demultiplexing and UMI clustering using only long-read data. Similarly, methods such as FLAMES [25] and Bambu [26] manage multiple stages of the analysis pipeline, ranging from read alignment and UMI deduplication to isoform quantification and discovery, while relying on external tools like BLAZE [27] and flexiplex [28] for barcodes and UMI identification. Furthermore, tools such as Isosceles [29] and IsoQuant [30] build upon established demultiplexing and UMI correction strategies to enhance transcript-level discovery and quantification. Recently, the *nf-core* community released a dedicated pipeline within the *nf-core* framework [31, 32], which integrates components such as BLAZE and IsoQuant for gene- and transcript-level quantification [33].

Yet, given the increasing applications of single-cell and spatial long-read sequencing in exploring transcriptome heterogeneity, evaluating and comparing these computational methods becomes crucial. Notably, bioinformatics software publications often present overly optimistic self-assessments [34, 35]. Thus, independent external benchmarking is essential to address these biases effectively [36, 37]. Additionally, accurate isoform detection is one of the key advantages of single-cell long-read sequencing technologies. While several isoform-level methods have been evaluated extensively in the context of bulk RNA-seq [38, 39], their performance on single-cell long-read data remains less explored.

In this study, we focused on Nanopore sequencing platforms to systematically benchmark state-of-the-art computational tools for single-cell and spatial long-read transcriptomics across four essential analytical dimensions. First, we assessed the number of detected UMIs and genes by comparing long-read approaches against short-read references. Second, we evaluated the barcodes identification and UMI correction and deduplication strategies, critical for accurate molecular identification and quantification. Third, we investigated the impact of these methods on transcriptomic profiling and cell annotation at the gene level. Lastly, we examined the performance of isoform detection, quantification, and discovery approaches. This comprehensive and independent benchmarking provides crucial insights for selecting optimal bioinformatics methods tailored to detect isoforms at the single-cell level.

## Materials and methods

An extended version of the ‘Materials and methods’ section is available in the Supplementary Methods.

### Data

#### Single-cell cDNA library preparation and sequencing

The MPNST1 and MPNST2 single-cell suspensions were converted into a barcoded scRNA-seq library with the 10x Genomics Chromium Single Cell 3′ Library, Gel Bead & Multiplex Kit and Chip Kit (v3). Short-read sequencing libraries were prepared following the 10x Genomics protocol then sequenced using an Illumina sequencer. Long-read sequencing libraries were prepared following the ScNaUmi-seq protocol [18] or the “Single Cell sequencing on Promethion protocol”, from Oxford Nanopore Technologies, with the SQK-PCS11 kit. Sequencing was performed on R9.4.1 flow cells at GenomiqueENS facility (MPNST1 on MinION, MPNST2 on a P2solo) and at the Centre National de Recherche en Génomique Humaine (CNRGH) facility (MPNST1 PromethION).

#### Spatial scRNA-seq long-read data

Spatial long-read data were downloaded from Gene Expression Omnibus (accession number: GSE153859), both as FASTQ and count matrix, according to the benchmarking steps. The subsequent processing was performed with the same pipeline as the nonspatial data.

#### Simulation of scRNA-seq long-read data

Simulated scRNA-seq long-read data were obtained with AsaruSim v1.0.3 [40], which simulates Nanopore datasets, closely mimicking real experimental data. It takes as input an isoform-by-cell count matrix to simulate reads. Using the real matrix as input therefore ensures that the transcriptomic profile of each cell is realistic at the isoform level. We used a subset of the real FASTQ files, derived from the R9.4.1 or R10.4.1 Nanopore chemistry, as a reference model to simulate realistic sequencing errors, alignment quality scores and coverage. The method to simulate multiple datasets with various UMI duplication rates is detailed in the Supplementary Methods. A comparison of the isoform length distribution between real and simulated data is shown in Supplementary Fig. S5C.

### Time and memory

To measure execution time and peak memory usage, reported in Supplementary Fig. S1, all jobs were executed on Linux servers equipped with Intel Xeon processors, 32 CPU cores, and 190 GB of memory. A maximum of 20 threads was allocated for all jobs. Execution time and maximum memory usage were assessed using the Linux time (*/usr/bin/time)* command with the *-v* flag. Execution time was extracted from the “Elapsed time” field, while peak memory consumption was recorded from the “Maximum resident set size” field.

### Definitions

We call **barcode** the 16-nucleotide sequence used to uniquely identify droplets or spots. All the possible sequences are referenced in an **inclusion list** (formerly called whitelist), provided by 10X Genomics. After sequencing, barcodes are generally compared to the inclusion list to discard unreferenced sequences. The sequenced barcodes found in the inclusion list correspond to the barcode **shortlist**. This shortlist is then further filtered to remove empty droplets/spots. A knee plot, depicting the number of UMI associated with each barcode from the shortlist, enables distinguishing empty from nonempty droplets. The barcodes associated with nonempty droplets are finally called **cell-associated barcodes**.

### Data processing

The tools and parameters associated with the short-read and long-read data are detailed in the Supplementary Methods. All software environments and version details are provided in Supplementary Table S3.

### Accuracy in barcodes and UMI assignment

#### Evaluation of barcode false-positive detection

Barcodes identified in long-read data but not present in the CellRanger-filtered shortlist were considered false positives, while true positives (TPs) were defined as barcodes present in both sets. For each long-read method, the precision–recall curve in Fig. 2B was made by computing the number of TPs across varying count thresholds, using the shortlist of barcodes identified by CellRanger on short-read data as a ground truth.

#### Accuracy of UMI error correction

To evaluate UMI correction accuracy, we compared the corrected and raw UMIs against the ground-truth UMI sequence from simulated data. Precision, recall, and F1-score were calculated using the following definitions. A TP was defined as a corrected UMI that matched the ground-truth UMI. Precision was computed as the proportion of correctly corrected UMIs out of all corrected UMIs (TP/total corrected), while recall was the proportion of correctly corrected UMIs out of all ground-truth UMIs (TP/total true). The F1-score was computed as the harmonic mean of precision and recall:


\begin{eqnarray*}
\mathrm{ F}1\ {\mathrm{ score}} = 2.\frac{{{\mathrm{ precision}}\ \times \ {\mathrm{ recall}}}}{{{\mathrm{ precision}}\ + \ {\mathrm{ recall}}}}
\end{eqnarray*}

In addition, clustering accuracy of UMI grouping was assessed using the Adjusted Rand Index (ARI), computed between the true UMI group labels and the predicted UMI clusters provided by each tool. All analyses were implemented in R using traceable R Markdown notebooks.

### Count matrix processing

#### Projection and cell type annotation

The count matrices were processed using Seurat’s functions (see Supplementary Methods for more details). Single cells were annotated for cell type using a modified version of Seurat’s AddModuleScore function and cell type-specific marker gene sets (see Code and Data availability).

#### Pseudo-bulk expression correlation analysis

Pseudo-bulk analyses were used for Fig. 2F, Supplementary Fig. S3G, and Fig. 5B. To compare pseudo-bulk expression profiles between methods, Seurat objects were annotated for cell types. Gene counts were aggregated across cells within each cell type to generate pseudo-bulk matrices. These matrices were normalized using counts-per-million followed by log-transformation. Pearson correlation coefficients were then computed between matched cell types across methods.

#### Quantitative assessment of cell type annotation and projection

To assess the quality of cell type annotation and the overlapping between integrated datasets on a projection, we used the local inverse Simpson’s index (LISI) [41], the ARI and the CHAOS score [42, 43].

#### Differential isoform analysis

Differential isoform analysis was performed using the Isoswitch R package (https://github.com/ucagenomix/isoswitch).

#### Gene ontology analysis

Gene ontology analysis was conducted on the differentially spliced genes (DSGs) using the clusterProfiler R package [44] and visualized using the enrichplot package.

### Genes and isoforms identification and quantification

We used the simulated data to assess the accuracy of transcript assignment.

#### Accuracy of reads assignment to isoforms

For each tool, transcript labels were parsed from the BAM files. Assigned transcript labels were compared to the simulated ground-truth transcript IDs. For each transcript class, precision and recall were computed using parallel processing across all simulation cycles. The F1-score was then calculated as the harmonic mean of precision and recall.

For real datasets, reconstructed isoform assemblies in GTF format generated by each pipeline were evaluated against the GENCODE M35 reference annotation using SQANTI3 v5.5.4, run in TUSCO mode with the “–tusco mouse” option. Precision and sensitivity metrics were extracted from the TUSCO report, and the F1 score was computed as:


\begin{eqnarray*}
\mathrm{ F}1\ {\mathrm{ score}}\ = 2.\frac{{{\mathrm{ precision}}\ \times \ {\mathrm{ sensitivity}}}}{{{\mathrm{ precision}}\ + \ {\mathrm{ sensitivity}}}}
\end{eqnarray*}

#### Comparison of gene and isoform expression estimation to ground truth

Each expression matrix (estimated and ground truth) was standardized using the mean and standard deviation derived from the ground truth. Then, the root mean squared error (RMSE) was calculated between the normalized matrices.

### Novel isoform prediction

#### Tool combination

For Isosceles and IsoQuant combination with preprocessing tools, combinations were restricted to technically compatible workflows. Since they require reads grouped by cell barcode and UMI stored in standardized CB/UB BAM tags, we combined them with outputs generated by Sicelore and wf-single-cell but not by FLAMES, scNanoGPS, or Bambu.

#### Discovery of novel isoforms in simulated data

A reduced reference annotation was built by excluding 15% of the expressed transcripts from the GENCODE M35 annotation. These 4412 transcripts were considered as the true novel isoforms. The incomplete reference annotation was provided to the isoform prediction tools and the GTP output has been parsed. Precision and sensitivity were computed, and the F1-score was calculated as the harmonic mean of precision and sensitivity.

#### Consistency among predicted annotations on real data

We used gffcompare to pairwise compare the predicted and the original annotations.

## Results

### Overview of the study

#### Benchmarking datasets

**Figure 1.**
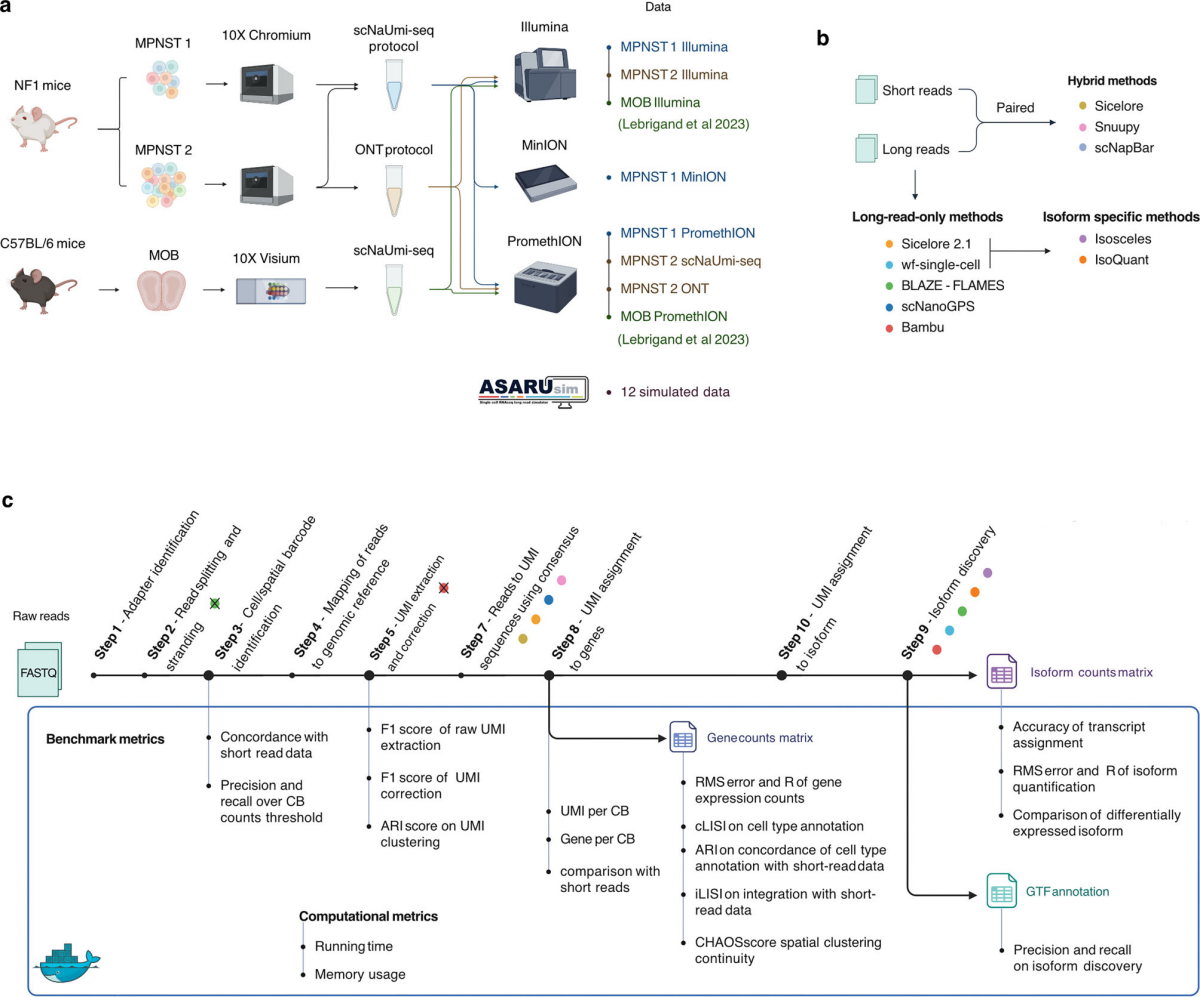
Overview of the benchmark. (**A**) This study was performed with a total of 20 datasets on various cell types and sample sizes. Six were newly-generated [malignant peripheral nerve sheath tumor (MPNST) 10X Genomics] covering two library preparation protocols and three sequencing platforms. Two were from a public dataset [mouse olfactory bulb (MOB), Visium], and 12 were simulated with the dedicated tool AsaruSim. (**B**) A total of 10 single-cell and spatial long-read RNA-seq methods were evaluated, organised into three categories: hybrid methods, long-read-only methods, and isoform-specific methods. (**C**) Data processing steps evaluated in this benchmark along with associated metrics. The preprocessing steps are organized chronologically, from raw FASTQ files to the generation of gene- and isoform-level count matrices. The step is supported by all methods, unless specified differently as follows: dots indicate the few methods supporting a particular step; crossed dots indicate the few methods that do not support a given step, with colors corresponding to the tools presented in panel (B). The entire benchmark is implemented as a reusable workflow in Nextflow and executed within Docker containers.

This benchmark was performed using 20 long-read Nanopore datasets: six novel scRNA-seq datasets generated with 10X Genomics technology, two publicly available spatial Visium RNA-seq dataset, and simulated scRNA-seq data (Fig. 1A and Supplementary Table S1). We selected the 10X Genomics technology, a droplet-based method, as it is the most used in the community, and the most mature in combination with long-read sequencing [13, 45].

The scRNA-seq data were newly-generated from MPNSTs developed by a mouse model of Neurofibromatosis Type 1 [46]; the two samples are designated MPNST1 and MPNST2. First, we performed short-read sequencing (Illumina), which yielded 491 and 401 million reads, respectively, with a mean of 107 700 and 38 900 reads per cell, ∼4500 and 10 000 single cells, and a total of ∼44 million and ∼134 million UMIs associated with genes, achieving high sequencing saturations of 81% and 34% for MPNST1 and MPNST2, respectively. We then sequenced the same MPNST1 samples with two long-read ONT platforms, enabling us to assess the impact of sequencing depth on methods performances. The MinION platform generated 11 million long reads, while the PromethION platform produced 151 million long reads, corresponding to sequencing saturations of 4% and 35%, respectively. The MPNST2 sample was solely sequenced on the PromethION platform, but following two distinct library preparation protocols: ScNaUmi-seq [18] and single-cell long-read ONT protocol (the ‘Materials and methods’ section), which produced 130 million reads and 108 million reads, respectively. These two datasets were included to evaluate the scRNA-seq long-read protocol differences in library and passed-read size characteristics on the same biological sample. The two MPNST2 long-read libraries showed only modest differences in cDNA library size and mean passed-read size (Supplementary Table S1), and no clear protocol-specific pattern was observed in the benchmark results.

Regarding the spatial long-read data, we downloaded a publicly available dataset (FASTQ files and gene count matrix of the corresponding short-read data), comprising ∼900 spatial spots from the olfactory bulb region of the mouse brain, generated using the Visium Spatial Gene Expression platform, and sequenced on ONT PromethION and Illumina [47].

To assess tool performance against a well-defined ground truth, we generated various simulated scRNA-seq long-read datasets using the simulation framework AsaruSim [40] (Supplementary Methods and Supplementary Table S1).

Altogether, these datasets constitute a valuable resource for benchmarking long-read single-cell transcriptomics methods, by varying both sequencing platforms (MinION versus PromethION), library preparation protocols (ScNaUmi-seq versus ONT), and R9.4.1 and R10.4.1 chemistry through simulations.

#### Selected bioinformatics tools and their specificities

We selected ten bioinformatics tools specialized in single-cell and spatial long-read transcriptomics through an extensive and systematic review conducted between 2022 and 2025, aiming to be as comprehensive as possible (Supplementary Table S2). Tools not included were either outdated, relying on custom tooling or *ad hoc* workflows, or lacked sufficient documentation. These tools were grouped into three categories (Fig. 1b). Hybrid approaches (Sicelore, Snuupy, scNapBar) rely on paired short-read data to guide barcode and UMI assignment. Long-read-only methods (Sicelore 2.1, wf-single-cell, Bambu, scNanoGPS, FLAMES-BLAZE) perform barcode demultiplexing, UMI clustering, and gene/isoform quantification directly from Nanopore reads, without requiring paired short-read data. Finally, isoform-specific methods (IsoQuant, Isosceles) build upon upstream demultiplexing strategies to enable reference-guided transcript discovery, quantification, and isoform-level analysis.

Together, these methods cover the main algorithmic strategies currently available for long-read single-cell and spatial data. Detailed descriptions of their implementations, parameter choices, and algorithmic specificities are provided in Supplementary Methods.

#### Benchmark and workflow

A comprehensive benchmark was conducted by applying a wide range of metrics to evaluate key steps of the preprocessing workflow, starting from barcodes identification up to the generation of gene and isoform count matrices (Fig. 1C). First, we compared their running time and memory usage, and tested their suitability for large datasets (Supplementary Results 1). Briefly, we observed variations among tools. In particular, scNanoGPS exhibited a longer execution time, Snuupy did not succeed in processing large datasets and scNapBar failed to produce results, even on small datasets. It was thus not further considered.

Then, we compared the number of UMIs and genes detected with paired short-read data. Subsequently, we evaluated the ability of methods to identify the cell-associated barcodes, extract and correct UMI tags. Next, we tested the fidelity of gene expression quantification at both single-cell and pseudo-bulk levels using simulated data. Then, we assessed the quality of cell type annotation obtained from real long-read data, as well as similarity and integration with short-read data. In addition, we evaluated the quality of spatial clustering by assessing the spatial continuity of the clusters and the similarity with clustering obtained from short-read data. Finally, we evaluated the ability of methods to correctly assign and quantify known isoforms, to discover novel ones, and to detect differential isoforms usage between cell clusters.

Our benchmarking workflow is provided as a reproducible Nextflow pipeline named scKeñver. It allows developers to test and evaluate new methods using our or their own datasets. All analyses were implemented in executable and traceable R notebooks. The computational reproducibility is enabled by using the Docker containerization technology [48].

### Comparison of the gene count matrices obtained from long-read and short-read scRNA-seq data

To evaluate the gene count matrices obtained after preprocessing the long-read datasets, we first compared, across tools, the number of UMIs and corresponding genes identified per cell or spot. Briefly, results showed that long-read-only approaches tend to detect a greater number of UMIs per cell, genes per cell, and total UMIs compared to hybrid approaches (Supplementary Results 2, Supplementary Fig. S2A, B, and D). We further evaluated the similarities between long-read and their counterparts short-read scRNA-seq count matrices. With all tools, we found relatively high average Pearson correlations of the number of UMI detected per gene, with a better performance for higher sequencing depth (Supplementary Results 2, Supplementary Fig. S2C). When comparing the number of UMI or genes detected per barcode, we observed high Pearson correlations for all methods, highlighting that long-read and short-read data are consistent at this level. Of note, lower correlations were found using FLAMES. This may partly reflect its lower UMI correction performance, although this effect does not fully explain inter-method differences, as final expression concordance also depends on other processing steps (Supplementary Results 2, Supplementary Fig. S2E).

### Accuracy in barcodes/UMI assignment and gene quantification fidelity

We evaluated the performance of the five long-read-only tools—BLAZE-FLAMES, Sicelore 2.1, wf-single-cell, scNanoGPS, and Bambu—in accurately identifying barcodes and correcting UMI sequences, as well as their fidelity and accuracy in quantifying gene expression at both the single-cell and pseudo-bulk levels. The barcodes identification consists in intersecting the barcodes inclusion list (formerly called whitelist), provided by 10X Genomics, with the set of the raw barcodes obtained from the sequencing. This intersection is called the shortlist and corresponds to potential cells/spots. From this shortlist, cell-associated barcodes are identified from the kneeplot.

#### Detection of cell-associated barcodes

**Figure 2.**
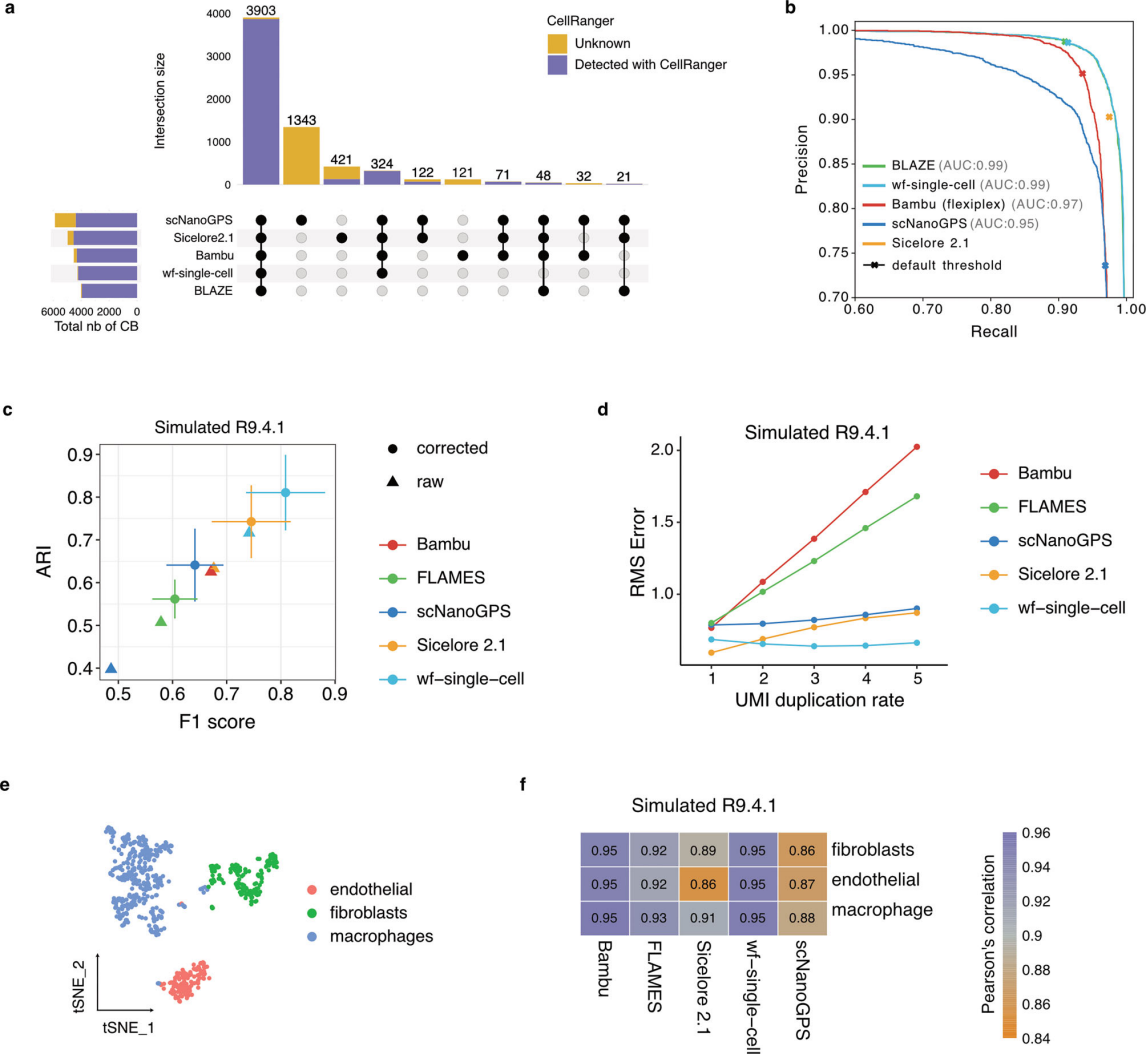
Barcode, UMI, and transcript identification. (**A**) Barcode upset plot comparing different cell-associated barcode lists. The bar chart on the left shows the total number of barcodes found by each tool. The bar chart on top shows the number of barcodes in the intersection of barcode lists from specific combinations of methods. The dots and lines underneath show the combinations. (**B**) Precision–recall curves across different barcode count thresholds. Precision and recall were calculated across different count thresholds by defining the barcodes identified from short reads as the ground truth, specifically the shortlist from Cell Ranger after the removal of empty droplets. Sicelore 2.1 is represented by a single point, as it does not implement barcode selection across varying count thresholds. (**C**) Scatter plot of the mean F1 score against mean ARI score for the UMI error correction. Error bars indicate the mean and standard error across simulated R9.4.1 datasets, on which methods run (*n* = 2; UMI duplication 1 and 5). Bambu does not perform UMI error correction, results are shown based on raw UMI only. (**D**) Comparison of gene expression estimation by different methods on the simulated R9.4.1 datasets (1 million reads each) across five different UMI duplication rates, evaluated by RMSE. (**E**) UMAP projection of gene expression level quantifications from ground truth of simulated data, colored by cell type. (**F**) Pearson’s correlation (color scale) of each method’s quantifications with expected quantification at pseudo-bulk level.

To assess their ability to detect cell-associated barcodes, we first compared the barcode list produced by each tool to the barcodes identified with CellRanger from the paired short-read data, on the MPNST1 PromethION dataset (Fig. 2A). In total, we identified 4513 cell-associated barcodes in the short-read MPNST1 dataset. Among these, 3903 barcodes (92%) were recovered by all long-read-only methods (Fig. 2a). Notably, scNanoGPS, Sicelore 2.1, and Bambu reported additional cell-associated barcodes not present in the short-read shortlist, respectively 1465 484, and 220 extra barcodes. Comparable performance patterns were observed in both the MPNST1 scNaUmi-seq (MinION) and MPNST2 ONT (PromethION) datasets (Supplementary Fig. S3A and B).

Since BLAZE, wf-single-cell, scNanoGPS, and Bambu infer the set of cell-associated barcodes by identifying an inflection point in the ranked barcodes count distribution (kneeplot), we computed precision–recall curves across varying barcode counts threshold to evaluate the false-positive detection rates of these approaches (Fig. 2B). As a ground truth reference, we used the barcode list generated from the short-read dataset by CellRanger, after filtering out empty droplets using the DropletUtils R package [49]. BLAZE and wf-single-cell showed the best performances (precision = 0.91; recall = 0.99 on default threshold) with a high consistency in the detection of cell barcodes, minimizing the false-positive. They are followed by Bambu (precision = 0.95; recall = 0.94 on default threshold). Although Sicelore 2.1 uses a different strategy to define the barcode shortlist, we compared its performance with the other methods. Unlike other long-read-only methods, Sicelore 2.1 does not rely on the inflection point of the ranked barcodes count distribution. Instead, it merges the sequenced barcodes with all possible 10X Genomics barcodes (inclusion list) within a defined Levenshtein distance. This strategy prioritizes a high recall (0.97) but leads to a slightly higher false-positive rate (precision = 0.9). The lowest performances were observed with scNanoGPS (precision = 0.74 and recall = 0.97 on default threshold) that assigns barcodes without any prior knowledge of the 10X Genomics barcode inclusion list.

#### Performance of the UMI error correction strategies

Next, we evaluated the performance of the UMI error correction strategies of each method. To obtain a reliable ground truth, we simulated single-cell long-read datasets using the AsaruSim tool [40] (the ‘Materials and methods’ section).

To assess the ability of each method to cluster UMIs originating from the same original transcript, we calculated the ARI score. Considering the R9.4.1 Nanopore chemistry, wf-single-cell exhibited the best UMI clustering performance, achieving a mean F1-score of 0.8  and the highest ARI score of 0.81. Sicelore 2.1 followed, with a mean F1-score of 0.74 and an ARI score of 0.74, while Bambu and FLAMES showed lower performance with F1-score 0.67 and 0.6 and ARI score 0.62 and 0.56 respectively (Fig. 2C, circle-shaped dots). Similar trends were observed with the R10.4.1 datasets, with an overall increased performance of the tools reaching F1-score of 0.95 and an ARI score of 0.98 (Supplementary Fig. S3C).

To further assess eventual variations in the efficiency of UMI sequence searching strategies, we compared the raw UMI sequence with the true UMI sequences and computed the same metrics (Fig. 2C and Supplementary Fig. S3C, triangle-shaped dots). For both R9.4.1 and R10.4.1 datasets, we observed significant differences among tools in terms of the quality of raw UMI sequences. wf-single-cell demonstrated the highest quality of raw UMI sequences (F1-score = 0.74; ARI = 0.71), whereas scNanoGPS exhibited the lowest UMI searching performance in R9.4.1 data (F1-score = 0.48; ARI = 0.39). The large difference in the quality of scNanoGPS raw and corrected UMI, especially in high error conditions (R9.4.1 datasets), suggests inferior UMI searching but good correction strategy. These findings suggest that the initial UMI sequence search and identification also plays a crucial role in achieving effective UMI deduplication.

#### UMI deduplication and gene expression quantification

Next, we investigated the impact of UMI deduplication on gene expression quantification. To this end, we compared the gene expression matrix generated by each tool to the expected (ground truth simulation) gene expression counts. To ensure consistent comparisons across tools and to reduce biases arising from library size, we applied a normalization step in which each matrix was standardized using the mean and standard deviation derived from the expected count matrix. Then, the fidelity of gene quantification across UMI duplication, measured as sequencing saturation, was assessed by computing the mean squared error (MSE) between estimated and expected gene expression values. Sequencing saturation reflects the fraction of reads that are UMI duplicates, i.e. originated from already-observed UMI. We found that increasing sequencing saturation leads to a gradual increase in gene expression estimation error for some methods (Fig. 2D). Notably, wf-single-cell showed the lowest estimation error across duplication rates (MSE = 0.66 at 5 UMI duplications), while FLAMES and Bambu exhibited substantial increases in MSE (exact values: MSE = 1.6 and 2.02, respectively, at 5 UMI duplications). Similar trends were observed in the R10.4.1 dataset, where wf-single-cell achieved the best estimation accuracy (MSE = 0.43 at 5 UMI duplications), followed by Bambu (MSE = 0.5 at 5 UMI duplications) (Supplementary Fig. S3D). Of note, Bambu performs UMI deduplication without a UMI error correction step. Cumulatively, its UMI deduplication relies on a fixed threshold for the number of UMIs, which makes its performance highly dependent on sequencing depth and read quality. This explains the sharp increase of performance at a 5 UMI duplication rate in the R10.4.1 simulated data (Supplementary Fig. S3D).

Overall, these results highlight the strong correlation between effective UMI error correction and accurate gene expression quantification. Indeed, tools with robust UMI correction—such as wf-single-cell and Sicelore—showed the most faithful gene-level estimates at high sequencing error rates. To support this observation, we simulated 20 million reads from 630 cells across three cell types—macrophages, endothelial cells, and fibroblasts—derived from the MPNST1 datasets, using two ONT error models (Supplementary Table S1, Simulated R9.4.1 and Simulated R10.4.1 datasets) (the ‘Materials and methods’ section). Overall, wf-single-cell yielded the best gene count estimations in both chemistries (MSE = 0.44 in R9.4.1 and MSE = 0.32 in R10.4.1), followed by Sicelore 2.1 (MSE = 0.63 in R9.4.1) and Bambu (MSE = 0.58 in R10.4.1). These results confirm Bambu’s marked performance gain specifically under the 5-UMI duplication condition in the R10.4.1 dataset (Supplementary Fig. S3E and F).

#### Overall gene expression correlation using pseudo-bulk data

Next, we investigated the gene expression at the pseudo-bulk level. We compared the estimated gene expression counts from each cell type with the true gene expression counts (the ‘Materials and methods’ section). Bambu and wf-single-cell achieved the best Pearson’s correlation for the three cell types (r = 0.95), followed by FLAMES (r = 0.93, 0.92, 0.92 for fibroblast, endothelial cells and macrophages, respectively) and Sicelore 2.1, achieving the lowest correlation (r = 0.91, 0.86, 0.89) (Fig. 2E and F). Similar results were observed with the R10.4.1 chemistry (Supplementary Fig. S3G).

### Evaluation of biological results at the gene level

#### In single-cell RNA-seq data

**Figure 3.**
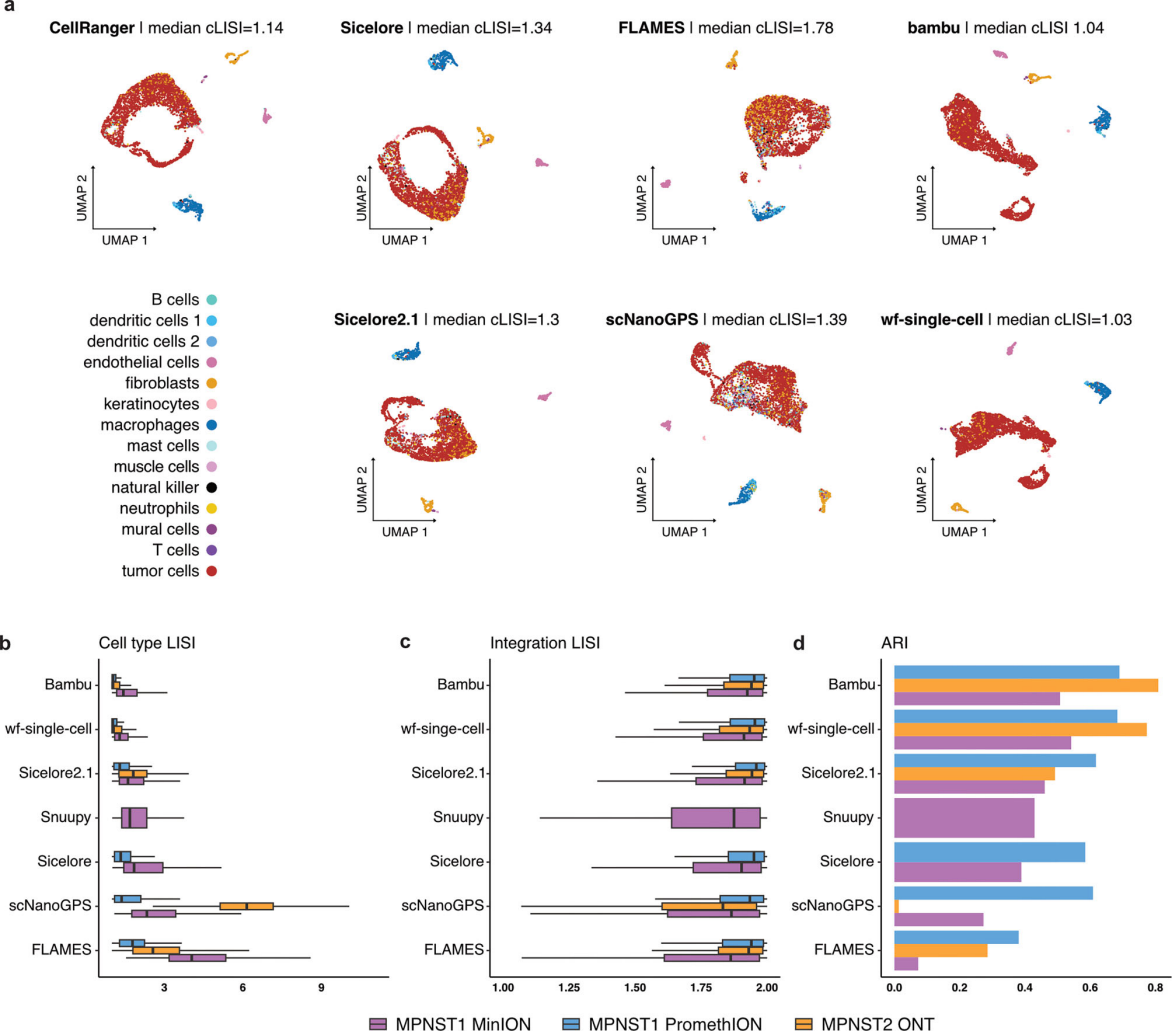
Biological results and comparison with short read data at the gene level. (**A**) UMAP representation of the MPNST1 PromethION dataset with cells colored by cell type annotation. The CellRanger UMAP visualization is based on short-read data, all other methods are based on long-read data. The median cell-type local inverse Simpson’s index (cLISI) is indicated at the top of each representation. (**B**) Quantitative assessment of cell type annotation performances in each method as measured by cLISI. Lower values indicate homogeneous annotation. (**C**) Quantitative assessment of alignment of UMAP representation of long-read versus short-read datasets as measured by integration local inverse Simpson’s index (iLISI). (**D**) Quantitative assessment of the concordance of long-read based with short-read based annotation cell type annotation measured by ARI score. Datasets used in panels (B)–(D) are color coded according to the legend below the panels.

To benchmark the effect of processing workflows on the downstream analysis results at the gene level, we first focused on the cell projection and cell type annotation with the MPNST1 PromethION dataset, on which long-read methods showed the best performances. As reference, the short-read sequencing of this dataset captured about 4500 cells, corresponding to 14 cell types. All gene count matrices preprocessed either by CellRanger for short-read data or by the tested tools for long-read data, were analysed similarly. After count matrix processing using Seurat V5, cells passing quality control steps were retained, annotated for cell types, and projected using UMAP (the ‘Materials and methods’ section). Regardless of the methods, the MPNST1 PromethION dataset contains four main populations, annotated as tumor cells (main population), macrophages, fibroblasts and endothelial cells (Fig. 3A).

We used an independent metric, the cLISI, to assess the quality of the cell-type annotation between short-read and long-read data. It quantifies the degree of local cell-type separation by combining the cell type annotation and the cell coordinates, with lower values indicating more homogeneous and consistent labeling [41]. wf-single-cell provided the most accurate cell-type characterization, both visually (Fig. 3A) and quantitatively, with a mean cLISI of 1.03 (Fig. 3B). It was followed closely by Bambu (1.04), then Sicelore 2.1 (1.30), Sicelore (1.37), scNanoGPS (1.39), and FLAMES (1.78), as measured on the MPNST1 PromethION dataset. Notably, both wf-single-cell and Bambu outperformed the short-read reference with this metric (median cLISI < 1.14). Consistent results were observed across MPNST1 MinION and MPNST2 ONT datasets (Fig. 3B).

To further compare long-read data processing by considering short-read data as reference, we merged each pair of long-read and short-read count matrices and generated a batch-effect corrected projection (the ‘Materials and methods’ section). Then, we computed two complementary metrics. The iLISI measures the degree of dataset mixing in a shared embedding space [41]. Secondly, the ARI assesses the consistency of cell-type annotations between the long-read and the short-read data. Bambu achieved the highest median iLISI and ARI scores across datasets (median iLISI = 1.94; ARI = 1.66), indicating better alignment and agreement with the short-read reference (Fig. 3C and D), followed by wf-single-cell (median iLISI = 1.93; ARI = 1.66) and Sicelore 2.1 (median iLISI = 1.94; ARI = 1.52). In contrast, FLAMES (median iLISI = 1.92; ARI = 1.24), and scNanoGPS (median iLISI = 1.90; ARI = 0.29) showed weaker agreement, with slightly lower integration and clustering consistency.

We further investigated some genes with expression detected in long-read but not short-read datasets. We observed that incomplete 3′-UTR annotations hampers the quantification of the 3′ signal in short-read data, resulting in missing or misquantified gene expression values (Supplementary Fig. S4). These biases can distort transcriptomic profiles and result in the misclassification of cell types. In contrast, as long-read sequencing is not restricted to 3′ end, it enabled accurate gene expression quantification despite incomplete 3′-UTR annotations, enhancing the cell type identification.

#### In spatial transcriptomics data

**Figure 4.**
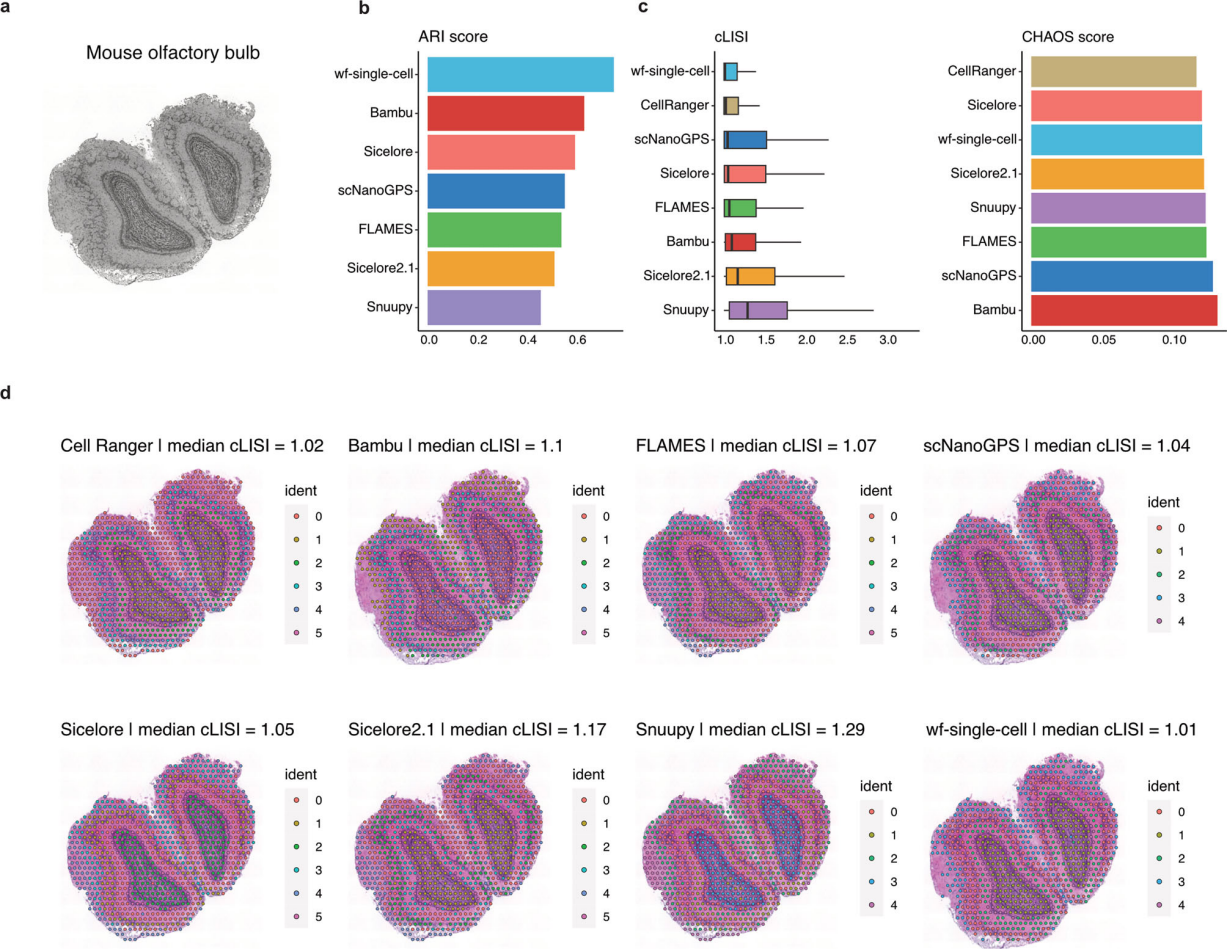
Comparison of spatial data clustering with short read data at the gene level. (**A**) Slice of the MOB, obtained by SpaceRanger, from Gene Expression Omnibus (accession number GSE153859). (**B**) Quantitative assessment of concordance of spatial clustering obtained with long-read (MOB PromethION) versus short-read (MOB Illumina) datasets, as measured by ARI score. (**C**) Quantitative measurement of the quality of spatial clustering using cLISI and CHAOS metrics, the lower the value, the more the method yields a continuous and homogeneous neighborhood for spatial spots. (**D**) Comparative visualization of clusters identified by long-read methods.

Regarding spatial transcriptomics, the Illumina dataset from the MOB, processed with the 10X Genomix Space Ranger pipeline, resulted in 916 spatial spots detected (Fig. 4A). Unsupervised clustering identified six clusters, consistent with the known anatomical regions of the olfactory bulb [47]. To assess gene-level performance in long-read methods, we preprocessed the MOB PromethION dataset with each method, and then performed similar unsupervised clustering of the detected spots. When comparing the clustering results to the short-read reference using ARI, wf-single-cell showed the highest similarity (ARI = 0.75), followed by Bambu (ARI = 0.63) and Sicelore (ARI = 0.59) (Fig. 4B). We further evaluated spatial clustering continuity using cLISI and CHAOS scores [38, 50], where lower values reflect more spatially coherent clusters. wf-single-cell again produced the most continuous and homogeneous spatial clusters (median cLISI = 1.01; CHAOS = 0.12), followed by Sicelore (cLISI = 1.05; CHAOS = 0.12) (Fig. 4c-d).

Together, these results demonstrate that long-read sequencing can achieve high-resolution clustering and accurate cell-type annotation, both in scRNA-seq and spatial transcriptomics data. Among the tested methods, wf-single-cell, Bambu followed by Sicelore consistently provided the most biologically meaningful clustering patterns, and strong performance in preserving biological signals during preprocessing.

### Evaluation of biological results at the isoform level

#### Isoforms characterization and quantification

**Figure 5.**
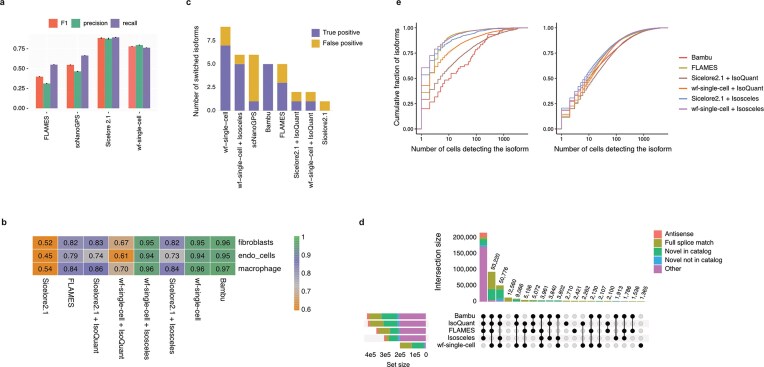
Accuracy of Isoforms characterization, quantification and discovery. (**A**) Accuracy of the tested tools to assign reads to annotated transcripts, as measured using precision, recall and F1 scores on the Simulated R9.4.1 dataset. Metrics are computed by comparing each transcript label assigned to reads with the labels of the ground truth. (**B**) Pearson’s correlation coefficient (color scale) of each method’s quantifications with the expected qualifications across three cell types, while considering pseudo-bulk in the R10.4.1 simulated data. (**C**) Barplot representing the number of differentially expressed isoforms between the three clusters identified by each tool in the simulated R9.4.1 dataset, classified as TPs (present in the expected list) or false positives (absent from the expected list) according to the ground truth. The ground truth was defined using the gene- and isoform-level count matrices generated by the AsaruSim simulation framework, from which a multiassay Seurat object was constructed. Note that Sicelore 2.1 + Isosceles did not detect any significant switched isoforms and is not represented on the barplot. (**D**) Upset plot showing the 20 largest intersections of isoform coordinates between isoform annotations identified by each tool in the real MPNST1 PromethION data. The bars are colored by isoform structural categories. (**E**) Cumulative fraction of novel (left) and known (right) isoforms as a function of the number of cells in which they are detected (*x*-axis, log scale) in the real MPNST1 PromethION data, for each tool or tool combination.

To benchmark transcript assignment accuracy, we evaluated the ability of each long-read-only method to correctly assign simulated long reads to annotated transcript models. To this end, we compared the annotated transcript model assigned to each read with its corresponding ground-truth transcript label in the Simulated R.9.4.1 dataset (Fig. 5A). Since Isosceles, IsoQuant, and Bambu assign transcript only after the UMI deduplication step, it was not possible to directly evaluate transcript assignment at the individual read level for these two methods. Sicelore 2.1 achieved the highest performance in transcript assignment (F1 score = 0.88), likely due to its ‘strict’ mode, which requires a complete exon-exon structure match for assignment. This was followed by wf-single-cell (F1 score = 0.78), while scNanoGPS and FLAMES showed more modest performance, with F1 scores of 0.54 and 0.39, respectively.

**Figure 6.**
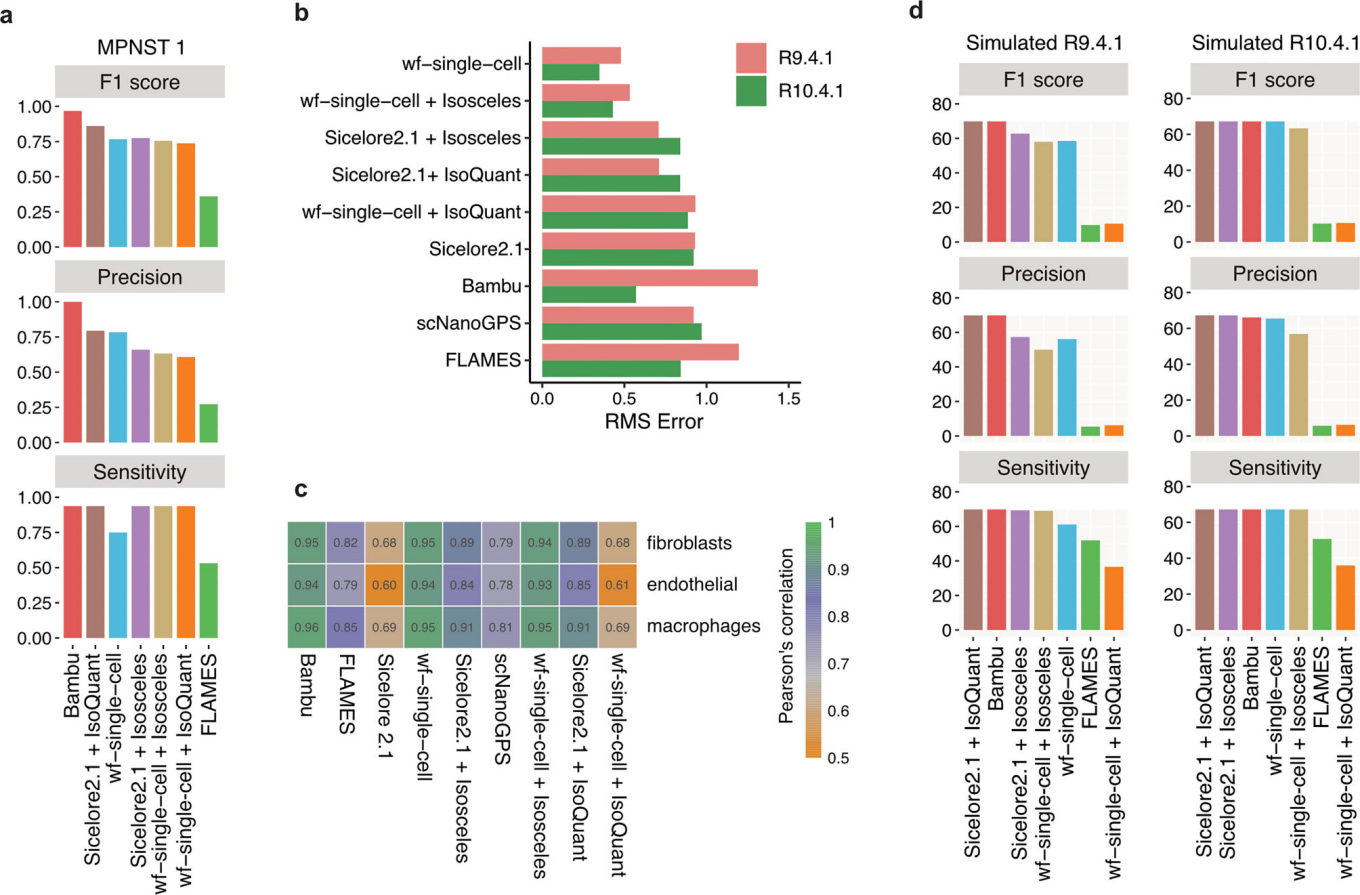
Isoforms characterization, quantification, and discovery. (**A**) Precision, sensitivity, and F1-score of known isoform assignment for each tool in the MPNST1 PromethION dataset, assessed using TUSCO genes as ground truth. (**B**) Ability to quantify transcripts measured using RMSE between the observed and expected (ground truth) transcript expression values. RMSE were computed after a normalization step, where each isoform count matrix was standardized using the mean and standard deviation of the ground truth. Both Simulated R9.4.1 and Simulated R10.4.1 datasets are shown. (**C**) Pearson’s correlation coefficient (color scale) of each method’s quantifications with the expected qualifications across three cell types, while considering pseudo-bulk in the R9.4.1 simulated data. (**D**) Precision, sensitivity, and F1-score rates of novel isoforms discovery by each tool in R9.4.1 and R10.4.1 simulated data.

We further examined isoform assignment accuracy in real datasets using a curated internal reference set of genes lacking alternative isoforms, the TUSCO genes [51]. TUSCO datasets assess the precision of long-read methods by identifying transcripts that deviate from reference annotations, and their sensitivity by evaluating the completeness of detection of known transcripts (Fig. 6A). Bambu, which leverages reference annotations to correct splice-junction alignments, demonstrates high precision (100%) and the best overall performance in detecting known isoforms, achieving an F1-score of 0.96. IsoQuant shows higher precision when coupled with Sicelore v2.1 (79%) compared with its combination with wf-single-cell (60%), highlighting the benefit of read-quality improvement achieved by Sicelore v2.1 through its consensus-based error-correction approach. In contrast, FLAMES exhibits the lowest performance across all three metrics (F1 score, precision, and sensitivity), indicating reduced accuracy in isoform assignment under these conditions. Overall, these results highlight substantial differences in isoform assignment accuracy among long-read analysis pipelines and underscore the importance of splice-junction correction and read-quality improvement for accurate isoform detection.

Next, we assessed the performance of these tools in transcript-level quantification by comparing the estimated transcript abundances with the expected values in the Simulated R9.4.1 and Simulated R10.4.1 datasets. As with gene expression analysis, we computed the RMSE after a normalization step, where each isoform count matrix was standardized using the mean and standard deviation of the ground truth (Fig. 6B). In both simulated datasets, wf-single-cell, either alone or coupled with Isosceles, achieved the most accurate isoform quantification, with the lowest RMSE values (0.48 and 0.53 in R9.4.1; 0.35 and 0.43 in R10.4.1, respectively). These were followed by Sicelore 2.1 coupled with Isosceles (RMSE = 0.71 in R9.4.1 and 0.84 in R10.4.1), while scNanoGPS and FLAMES exhibited the highest deviations, with RMSE values of 0.92 and 1.2 in R9.4.1, and 0.97 and 0.84 in R10.4.1, respectively. These results consistently demonstrate the high fidelity of wf-single-cell in quantifying known transcript isoforms across both sequencing chemistries.

Transcript quantification was further evaluated at the pseudo-bulk level by aggregating isoform counts across cells within the three simulated cell types. Overall, we observed a high correlation between the resulting expected and observed transcript abundances (Fig. 6C), supporting the ability of long-read methods to reliably quantify isoforms at the population level. Bambu exhibited the highest concordance with the ground truth, with Pearson correlation coefficients of 0.95, 0.94, and 0.96 for fibroblast, endothelial cells, and macrophage populations, respectively. This was followed by wf-single-cell and Isosceles, which also showed strong correlations. In contrast, Sicelore 2.1 demonstrated the lowest correlation scores among the evaluated methods. Similar trends were observed in the R10.4.1 dataset (Fig. 5B), further confirming the consistency of these findings across different sequencing chemistries.

#### Differential isoform analysis

Next, we investigated genes affected by differential isoform usage between cell types with the Isoswitch R package that leverages the Seurat’s FindMarkers function to identify isoform switches between each possible pair among the three cell populations of the simulated datasets. DSGs were filtered using an adjusted p-value threshold of 5%. We then compared the list of DSGs from long-read methods to the ground truth (Fig. 5C and Supplementary Methods). Bambu led in performance, identifying 6 out of 18 DSGs confirmed by the ground truth. wf-single-cell combined with Isosceles followed closely with five confirmed isoforms and one false positive, and FLAMES identified 3 out of 18 DSGs. In contrast, Sicelore 2.1 alone did not detect any confirmed DSGs.

**Figure 7.**
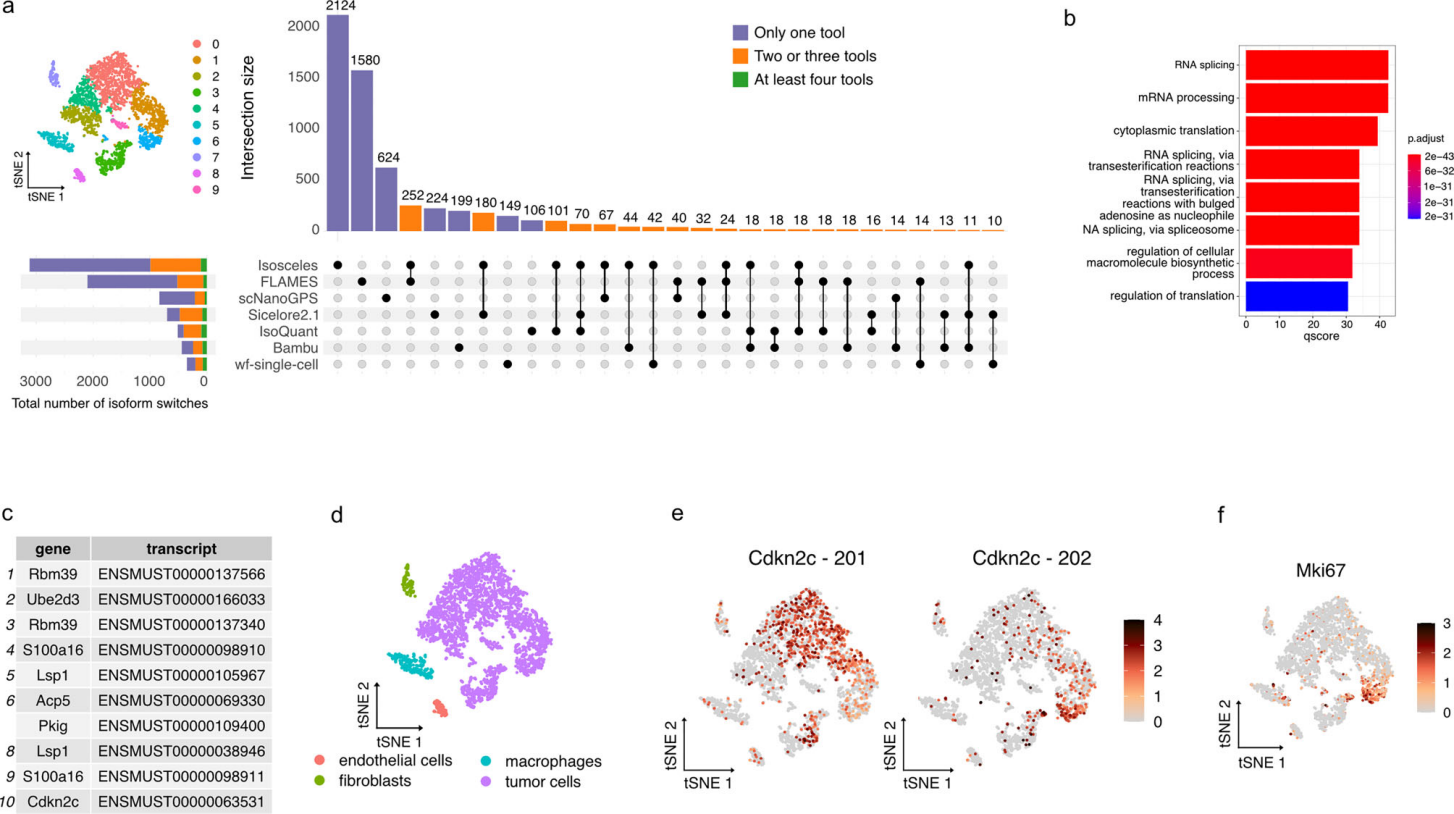
Comparison of the genes showing an isoform switch, depending on the preprocessing tools. (**A**) (left) A t-distributed stochastic neighbor embedding (tSNE) projection of the MPNST1 PromethION dataset at the gene level. Cells are colored according to 10 cell clusters. (right) Upset plot comparing the number of isoforms characterized by a switch between any two cell clusters, for each preprocessing tool. The bars are colored according to the number of tools having identified the isoforms. In total, 6266 isoforms were identified characterized by a switch, corresponding to 2942 genes. (**B**) Barplot showing the top 8 enriched gene ontologies associated with the 2942 genes characterized by an isoform switch. (**C**) Top 10 genes showing an isoform switch between any two cell clusters, displayed with their Ensembl transcript identifier. (**D**) tSNE plot with cells colored according to four cell types. (**E**) tSNE plot depicting the isoform switch of *Cdkn2c* gene, with differential usage of the isoforms 201 and 202 of this gene. (**F**) tSNE plot depicting the expression of the *Mki67* gene, used as a proxy for proliferative activity.

We then analysed the real MPNST1 PromethION dataset to evaluate the effect of each preprocessing tool on the final detection of DSGs between cell clusters. This analysis was performed on each isoform count matrix with Isoswitch, between each pair of the 10 cell clusters, to identify the genes showing an isoform switch between two cell clusters (Fig. 7A). Few isoforms are detected consistently by four tools (green bars). We noticed that tools such as Isosceles, FLAMES and scNanoGPS detect more switched isoforms than other tools, which would require customized in-depth analyses to determine if they are false positives or not. To investigate the biological functions behind the detected isoform switch, we performed gene ontologies analysis using the 2942 genes detected by all methods (Fig. 7B). Genes characterized by isoforms switches are mostly related to the processing of RNA molecules, suggesting that they are themselves directly related to the splicing process. Among the top ten genes characterized by isoforms switches, consistently detected by at least six tools, we pinpointed *Cdkn2c*, as in this biological context, *Cdkn2a is* known to be deleted [52] (Fig. 7C). Additionally, other isoforms switches concerned variations between cell types, the isoform switch of *Cdkn2c* was restricted to the tumor cell population (Fig. 7D and E). We observed that the expression of the isoform 202 coincides with a proliferative cell activity (Fig. 7F), while the isoform 201 is over-expressed in quiescent cells. This observation is consistent with observations made in T cells in physiological conditions [53]. Recently, AS of *Cdkn2c* has been shown to be correlated with distinct response to treatment in the context of ovarian cancer [54].

#### Isoforms discovery

Among the evaluated tools, wf-single-cell, FLAMES, Bambu, Isosceles, and IsoQuant support transcript discovery on their own, i.e. novel transcripts not part of the annotation (Supplementary Table S2). Yet, Isosceles and IsoQuant are isoform-specific tools (Fig. 1B), and thus require preprocessing by other tools such as wf-single-cell and Sicelore (see the ‘Materials and methods’ section). We compared their performances on the Simulated R9.4.1 and Simulated R10.4.1. Transcript discovery was evaluated by randomly removing 4412 (15%) expressed isoforms-defined as isoforms with nonzero UMI counts-from the GENCODE annotation, to mimic real-world scenarios where unannotated transcripts are present (the ‘Materials and methods’ section). This reduced annotation was then provided as the reference for each tool, and their ability to recover the removed transcripts as novel was assessed using gffcompare [55] (Fig. 6D). The set of predicted novel transcripts from each tool was compared to the set of intentionally removed isoforms to evaluate transcript discovery performance.

Compared to most tools, Bambu alone yields highly accurate novel transcript models on R9.4.1 data, likely due to its splice alignment correction strategy, achieving an F1-score of 73%. Isosceles demonstrates slightly higher precision (57%) when coupled with Sicelore 2.1 compared to its combination with wf-single-cell (50%), while maintaining a comparable sensitivity (69%) in both cases. Isosceles combined with Sicelore 2.1 reports 1083 (15%) false positive exons, compared to 2361 (14%) when used with wf-single-cell. This difference is likely attributable to splice site alignment errors, suggesting improved accuracy when using the consensus-based correction methods employed by Sicelore 2.1. On the R10.4.1 dataset, Isosceles slightly outperformed other methods, achieving an F1-score of 70%, compared to 68.5% for Bambu, 68% for wf-single-cell, and 63% for Isosceles coupled with wf-single-cell. In contrast, FLAMES showed poor recall across both datasets, highlighting its limitation in novel isoform detection.

To examine transcript assembly on a real dataset, where the ground truth is unknown, we compared the coordinates of all detected isoforms with the known transcripts from the reference annotation. On the MPNST1 PromethION dataset, the highest percentage of known transcripts, represented by the ‘full splice match’ isoform structural category detected by gffcompare, was achieved by wf-single-cell (79 986 transcripts; 46%) (Fig. 5D). The number of novel transcripts, represented by all ‘novel’, and ‘other’ categories, shows the highest differences. While wf-single-cell had the lowest number of ‘other’ isoform structural categories (4%), a very high percentage of novel transcripts were detected by Bambu (262 175 transcripts; 93%) and IsoQuant (256 260 transcripts; 77%). Regarding the intersection between annotations, Bambu and IsoQuant achieve the highest number of isoforms confirmed by at least three other tools (363 755 and 359 810 isoforms), wf-single-cell achieves the lowest number (147 973 isoforms). Similar trends are observed regarding only isoforms reported as novel (Fig. 5D). The high number of novel transcripts detected by Bambu and IsoQuant in real data likely reflects increased discovery sensitivity, but may also be amplified by sparse expression and transcript-model expansion effects that are not fully represented in the simulated benchmark.

To further characterize these novel isoforms, we examined the number of cells in which each isoform was detected, and compared this distribution with that of known isoforms (Fig. 5E). As expected, novel isoforms were generally detected in fewer cells than known isoforms. On average across methods, 75.6% of novel isoforms were detected in at most 10 cells, compared with 50.8% of known isoforms, and 90.6% of novel isoforms were detected in at most 100 cells, compared with 82.9% of known isoforms. Consistent with this overall trend, ∼53% of novel isoforms identified by Isosceles were detected in a single cell, compared with 36%, 29%, and 20% for FLAMES, IsoQuant, and Bambu, respectively, whereas only 5% (Isosceles) and 3% (FLAMES) were detected in at least 100 cells, compared with 16% and 20% for IsoQuant and Bambu. These results show that low cell-frequency is enriched among novel isoforms, but is also common among known isoforms in single-cell data. Therefore, isoforms detected in only a few cells may correspond to false-positive calls, incompletely processed transcripts, or true rare isoforms associated with small cellular subpopulations.

## Discussion

We benchmarked 10 single-cell and spatial long-read tools using several datasets generated with different sequencing platforms, protocols, and chemistries. We used several metrics that measure quality control and concordance with short-read data, including the accuracy in barcodes/UMI assignment, the fidelity of gene and transcript expression quantification, the clustering, and cell type identification at single-cell and spatial level as well as the isoforms characterization and quantification. Overall, tools that used only long-read data performed well across datasets, suggesting a paired short-read dataset is not necessary anymore. We observed that method performance is dependent on sequencing depth and sequencing quality. For instance, except snuupy which failed to process PromethION data, most tools perform better with PromethION data than MinION data, and with the R10.4.1 rather than with the R9.4.1 chemistry. The performance also depends on the task. For example, wf-single-cell performs better in the gene expression quantification, but is surpassed by Bambu and Isosceles at isoform discovery. In contrast Bambu misidentifies UMI sequences leading to an overestimation of RNA counts.

Unexpectedly, long-read methods, particularly Bambu and wf-single-cell, outperform CellRanger in the characterization and annotation of cell types. This is likely due to the 3′ bias in short-read data, which can compromise accurate gene expression detection. In line with this, Bambu and wf-single-cell consistently detect a greater number of genes per cell than CellRanger. Similarly, when assessing concordance with short-read data, a discrepancy emerges: tools that show the highest correlation with short-read datasets do not necessarily perform best on simulated data. This further supports recent findings [39, 56, 57] showing that short-read data are not free from bias, reinforcing the value of long-read approaches for transcriptomic profiling.

We observed a consistent relation between UMI error correction and the accuracy of gene and transcript expression quantification. For instance, wf-single-cell employs an advanced UMI error correction strategy based on a directed graph algorithm, resulting in the highest fidelity in gene/transcript expression estimation. In contrast, Bambu does not perform UMI error correction, which leads to erroneous UMI deduplication and consequently an overestimation of expression counts—particularly under conditions of high sequencing saturation and high sequencing error rates. Notably, this overestimation strongly correlates with the expected expression levels.

The higher sequencing error rate observed in long-read data, compared to short-read sequencing, adversely affects the accuracy of read alignment, particularly at splice junctions [58]. To overcome this limitation, error correction approaches such as consensus sequence generation, as implemented in Sicelore, or splice alignment correction, as adopted in Bambu, can be applied [59]. Consistent with this, our results showed that Sicelore 2.1 achieves more accurate read assignment to annotated transcript models, while Bambu provides the most reliable transcript assembly under high-error condition. Moreover, coupling wf-single-cell with Isosceles or IsoQuant tends to predict more false-positive novel transcript models, while coupling Sicelore 2.1 with Isosceles or IsoQuant results in splice alignment errors.

While gene- and transcript-level quantification were globally consistent across several methods, differential isoform usage analyses remained markedly method-specific, suggesting that local isoform-switch calls are more sensitive to sparsity and transcript modeling uncertainty than aggregate expression estimates.

These observations highlight limitations of current benchmarking strategies for single-cell long-read transcriptomics, as good global quantification concordance does not necessarily translate into consistent DSG detection across methods. They further emphasize the need for improved evaluation metrics for differential splicing analyses and more robust ground-truth strategies for novel transcript and isoform-level benchmarking.

Despite the rapid development of single-cell and spatial long-read RNA-sequencing methods, setting up unbiased and objective benchmarking remains challenging. A major barrier to systematic benchmarking is the lack of standard output formats. Specifically, we faced issues to automate the parsing of the count matrices. Indeed, depending on the tools, matrices are either stored in sparse or dense format, with or without the presence of metadata, such as exon sizes. Overcoming the technical challenges of output formats would enable the introduction of continuous and automated comparisons across tools.

In contrast to bulk and single-cell short-read RNA-seq, for which extensive public data for benchmarking are available [56, 60–62], single-cell and spatial long-read transcriptomics still lacks comprehensive benchmark resources. This represents a substantial obstacle to optimize long-read methods for transcriptome profiling. Although long-read RNA-seq datasets are increasingly available for bulk experiments [39, 45, 59], resources for single-cell and spatial contexts remain limited. In this study, we aimed to address this gap by providing access to a collection of datasets specifically generated across different sequencing protocols, depths, and chemistries. However, our study relies on the mouse model in the specific contexts of MPNST and olfactory bulb, which limits the extrapolation of our benchmark to other applications. Another limit of our study is the focus on 3′ 10x Genomics library preparation, while 5′ library is not tested [63]. Finally, our study is restricted to Nanopore, and not other long-read platforms such as PacBio [13]. Our conclusions cannot be broadened to these other types of data, despite being supported by several of the tools evaluated in this study.

One of the key advantages of long-read RNA-seq is its ability to identify fusion transcripts or variants in single-cell and spatial contexts. Yet, most of the reads are not full-length, and thus do not recapitulate the annotated isoforms in the reference transcriptome (Supplementary Fig. S5). This observation has already been reported in other studies [63, 64].

Despite the importance of these features, our benchmark focused on core transcriptomic tasks, such as UMI/barcode detection, gene and isoform quantification, clustering, and annotation due to the current lack of standardized tool outputs and methods for evaluating fusion detection and variant calling in these settings.

Altogether, this study brings three main contributions: (i) a collection of single-cell long-read datasets specifically designed to support the benchmarking of bioinformatics tools, (ii) an independent evaluation of ten computational methods providing a practical resource to select the most appropriate tools according to the desired analysis (Supplementary Fig. S6), and (iii) a reusable benchmarking workflow enabling method developers to compare their own approaches against the set of reference methods evaluated in this work.

## Supplementary Material

lqag070_Supplemental_File

## Data Availability

Sequencing data related to the MPNST1 and MPNST2 samples are available on ArrayExpress, under accession number **E-MTAB-15190**. It includes the FASTQ files and the count matrices obtained with the wf-single-cell pipeline. The workflow behind the benchmark, called scKeñver, is available on Github (https://github.com/alihamraoui/scKenver). The specific version used here is available on Zenodo (https://doi.org/10.5281/zenodo.17312868). This repository further contains the data simulated with AsaruSim and the gene markers used for cell type annotation.
