# Supplementary material for: A systematic benchmark of bioinformatics methods for single-cell and spatial RNA-seq nanopore long reads data
Source: NAR Genom Bioinform. 2026 Jul 6;8(3):lqag070. doi: 10.1093/nargab/lqag070 (PMC13335474; doi:10.1093/nargab/lqag070)
Supplement: lqag070_Supplemental_File [file lqag070_supplemental_file.pdf]

Supplementary Material to

A systematic benchmark of bioinformatics methods for single-cell and spatial RNA-seq Nanopore long reads data

Ali Hamraoui[1,2], Audrey Onfroy[3], Catherine Senamaud-Beaufort[1], Fanny Coulpier[3], Sophie Lemoine[1], Laurent Jourdren[1], Morgane Thomas-Chollier[1,2]

1. GenomiqueENS, Institut de Biologie de l'ENS (IBENS), Département de biologie, École normale supérieure, CNRS, INSERM, Université PSL, 75005 Paris, France
2. Group Bacterial infection, response & dynamics, Institut de biologie de l'ENS (IBENS), École normale supérieure, CNRS, INSERM, Université PSL, 75005 Paris, France
3. Team Neurofibromatosis and Lymphoma oncogenesis, Institut Mondor de Recherche Biomédicale, UPEC, INSERM, 94000 Créteil, France

Distributed under a Creative Commons Attribution | CC-BY 4.0 International license

Supplementary Results 3
1. Running time and memory usage 3
2. Comparison of the gene count matrices obtained from long-read and short-read scRNA-seq data 3
Supplementary Figures 6
Figure S1 | Running time and memory usage. 6
Figure S2 | Comparison of gene count matrices obtained from long-read versus short-read data. 7
Figure S3 | Accuracy in barcode assignment and gene quantification fidelity. 8
Figure S4 | Importance of gene annotation for isoform detection. 9
Figure S5 | Read length distributions of ScNaUmi-Seq PromethION data for MPNST1 10
Figure S6 | Decision-tree guidelines for scRNA-seq long-read data analysis. 11
Supplementary Tables 12
Table S1 | Datasets used in this study. 12
Table S2 | Ten scRNA-seq long-read methods evaluated in this study. 13
Table S3 | Software version and environment control strategies 14
Supplementary Methods 15
Selected bioinformatics tools 15
Data 16
Single-cell cDNA library preparation and sequencing 16
Generation of single-cell cDNA 16
Short-read Illumina libraries 16
Long-read ScNaUmi-seq protocol libraries 16
Single-cell long-read ONT protocol libraries 16
Spatial scRNAseq long-read data 16
Simulation of scRNAseq long-read data 17
Time and memory 18
Definitions 18
Data processing 18
Tools and parameters associated with short-read data 18
Tools and parameters associated with long-read data 18
Accuracy in barcodes and UMI assignment 19
Evaluation of barcode false-positive detection 19
Accuracy of UMI error correction 20
Count matrix processing 20
Projection and cell type annotation 20
Pseudo-bulk expression correlation analysis 21
Quantitative assessment of cell type annotation and projection 21
Differential isoform analysis 22
Gene ontology analysis 22
Genes and isoforms identification and quantification 22
Accuracy of reads assignment to isoforms 22
Comparison of gene and isoform expression estimation to ground truth 23
Novel isoform prediction 23

Distributed under a Creative Commons Attribution | CC-BY 4.0 International license

Discovery of novel isoforms in simulated data 23
Consistency among predicted annotations on real data 24
References **25**

Supplementary Results

1. Running time and memory usage

We compared the runtime and memory usage of the ten evaluated methods (Figure S1), which showed differences between hybrid, long-read-only and isoform-specific approaches.

While the two hybrid methods, Snuupy and Sicelore, required respectively 25 and 11 hours to process 10 millions reads, almost all long-read-only methods took less than 4 hours. Exception is made by scNanoGPS, which exhibited a remarkably longer runtime than other long-read-only methods (20h25min), primarily due to its barcode curation step (16h58min with 30 threads) (Figure S1a). Moreover, the curation step of scNanoGPS is heavily influenced by the size of the barcode library: 9h23min for 900 cell-associated barcodes vs 34h41min for 4500 cell-associated barcodes for a total of 1 million reads in both datasets (Figure S1b). Regarding memory usage, the three long-read-only methods FLAMES, wf-single-cell and Bambu demonstrated remarkable memory efficiency compared to hybrid methods. However, the amount of memory used by Sicelore 2.1 and scNanoGPS was comparable to hybrid methods (Figure S1c). Concerning isoform-specific methods, IsoQuant outperformed Isosceles both in terms of running times (15 min versus 57 min to process 10 millions reads) and memory usage (26 MB versus 8GB for 10 millions reads).

Considering scalability, Snuupy did not succeed in processing the PromethION datasets, as it required up to 200 GB of RAM for BLAST step. This poor scalability makes it unsuitable for large-scale datasets, without improvements. Similarly, scNapBar failed to produce results with MinION datasets (about 10 million reads) even after 120 hours of running. It was thus not tested on other datasets and was excluded from subsequent evaluations.

2. Comparison of the gene count matrices obtained from long-read and short-read scRNA-seq data

To evaluate the gene count matrices obtained after preprocessing the long-read datasets, we first assessed the number of UMIs and corresponding genes identified per cell or spot across methods. The raw gene expression matrix output of each method was used to quantify (i) the number of UMIs per barcode, (ii) the number of genes detected per barcode (genes with non-zero expression), and (iii) the total number of UMI counts in the matrix. These metrics were then compared to short-read data.

Distributed under a Creative Commons Attribution | CC-BY 4.0 International license

Furthermore, we compared the gene expression for each barcode between short- and long-read data (Figure S2).

Distinct trends emerged across the computational tools, particularly in the total number of UMIs detected, and consequently in the number of UMIs per barcode. Long-read-only approaches tend to detect a greater number of UMIs compared to hybrid approaches (Figure S2a,d), which may reflect both increased sensitivity and/or a higher rate of false positives. To distinguish between these two cases, we used the short-read dataset as a reference. With respect to UMI and gene detection per cell, Bambu showed a slight increase in UMI counts while maintaining high correlation with short-read data, suggesting greater sensitivity in UMI detection (Figure S2a–c). The elevated number of detected genes in Bambu can also be attributed to its ability to predict novel genes, contributing to its expanded gene detection profile.

We then compared gene expression with short-read data using Pearson's correlation. Overall, we found relatively high average Pearson correlations with all tools (Figure S2c). We observed consistent trends across data types and sequencing depth. To evaluate the effect of sequencing depth, we compared the same sample sequenced on high- versus low-throughput platforms. Notably, all workflows performed better with higher sequencing depth (MPNST1 PromethION data) compared to lower sequencing depth (MPNST1 MinION data), attesting a depth sensitivity. In contrast to the scRNA-seq datasets, which exhibited high median Pearson’s correlation coefficients, the spatial transcriptomics data showed substantially lower correlations. Specifically, Pearson’s correlation coefficients ranged from r=0.4 for FLAMES to r=0.75 for Snuupy, indicating poor concordance. These values are even lower than those observed with the MinION scRNA-seq data, despite its lower sequencing depth and saturation. These results are inherent to this spatial transcriptomics dataset, which combines a lower number of UMI per spot, a high sequencing saturation and sequencing errors, thereby resulting in UMI scattering with inflated UMI counts (1). This phenomenon further leads to lower correlations with the short-read datasets.

The same conclusions are reached when comparing the number of UMI and genes detected within each cell, between long-read and short-read data (Figure S2). High Pearson correlations were observed for all methods, highlighting that long-read and short-read data are consistent to evaluate the biology of cells. By comparing computational methods, lower correlations are found using FLAMES. This can be explained by the UMI correction issues, associated with sequencing errors. By comparing sequencing library preparation protocols, lower correlations are found when evaluating the number of UMI per spot, in spatial data, using the long-read-only methods (Figure S2e). Coherently, regardless of the protocol, correlations are higher when considering hybrid methods than long-read-only methods. This is expected, as, by definition, hybrid methods rely on paired short-read data for UMI assignment. However, although correlations are high, hybrid methods tend to detect less UMI and genes per cell. Similarly, when comparing sequencing platforms, we observed underestimation of both the number UMI and the number of genes per cell using MinION, compared to PromethION. This can be related to the lower sequencing depth associated with MinION flowcells.

Distributed under a Creative Commons Attribution | CC-BY 4.0 International license

Supplementary Figures

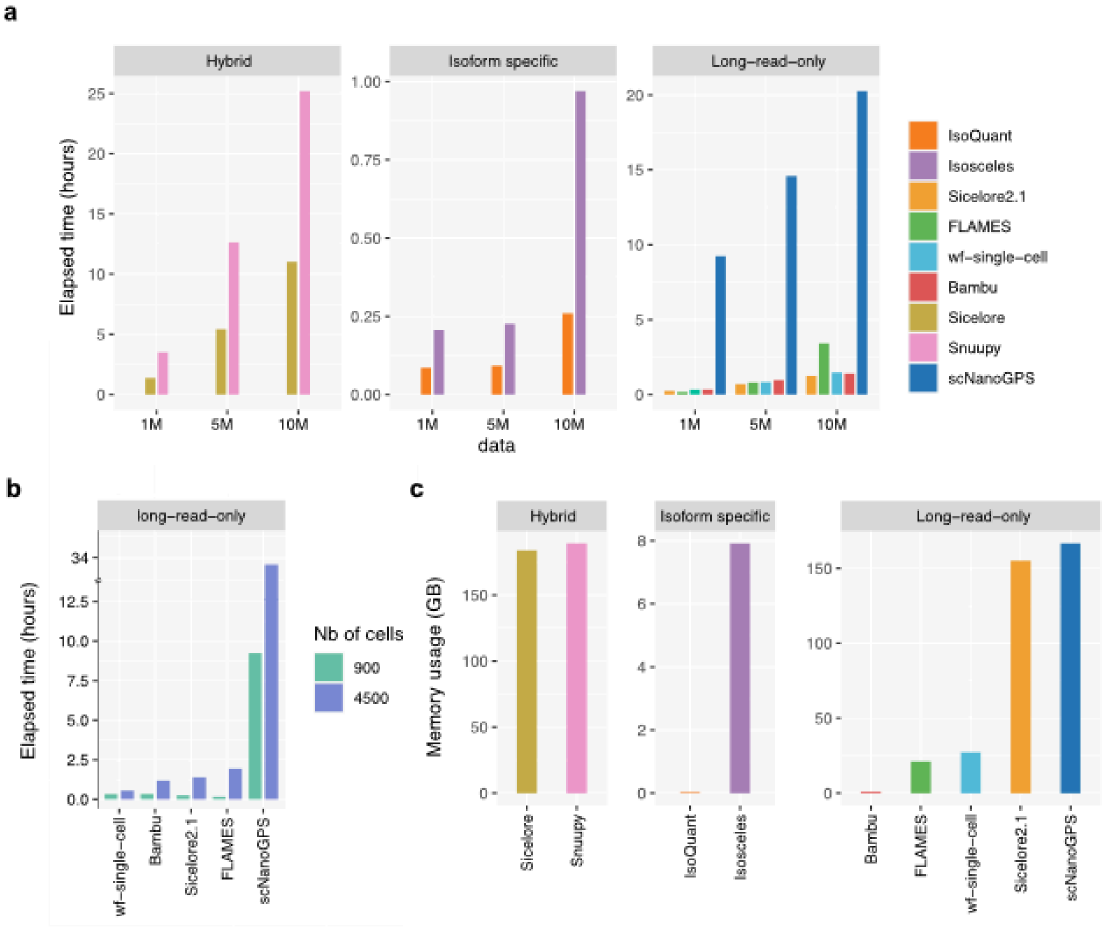

Figure S1 | Running time and memory usage.

(a) Elapsed time in hours for hybrid, long-read-only and isoform specific methods computed for 1, 5 and 10 million reads obtained by subsampling the MOB datasets. scNapBar is not included due to exceeding running time. **(b)** Elapsed time in hours for long-read-only methods computed for 1 million reads from 900 cells of the MOB datasets versus 1 million reads from 4500 cells in simulated datasets. **(c)** Peak of memory used by hybrid, long-read-only and isoform specific methods used for 10 millions reads from the MOB datasets.

Distributed under a Creative Commons Attribution | CC-BY 4.0 International license

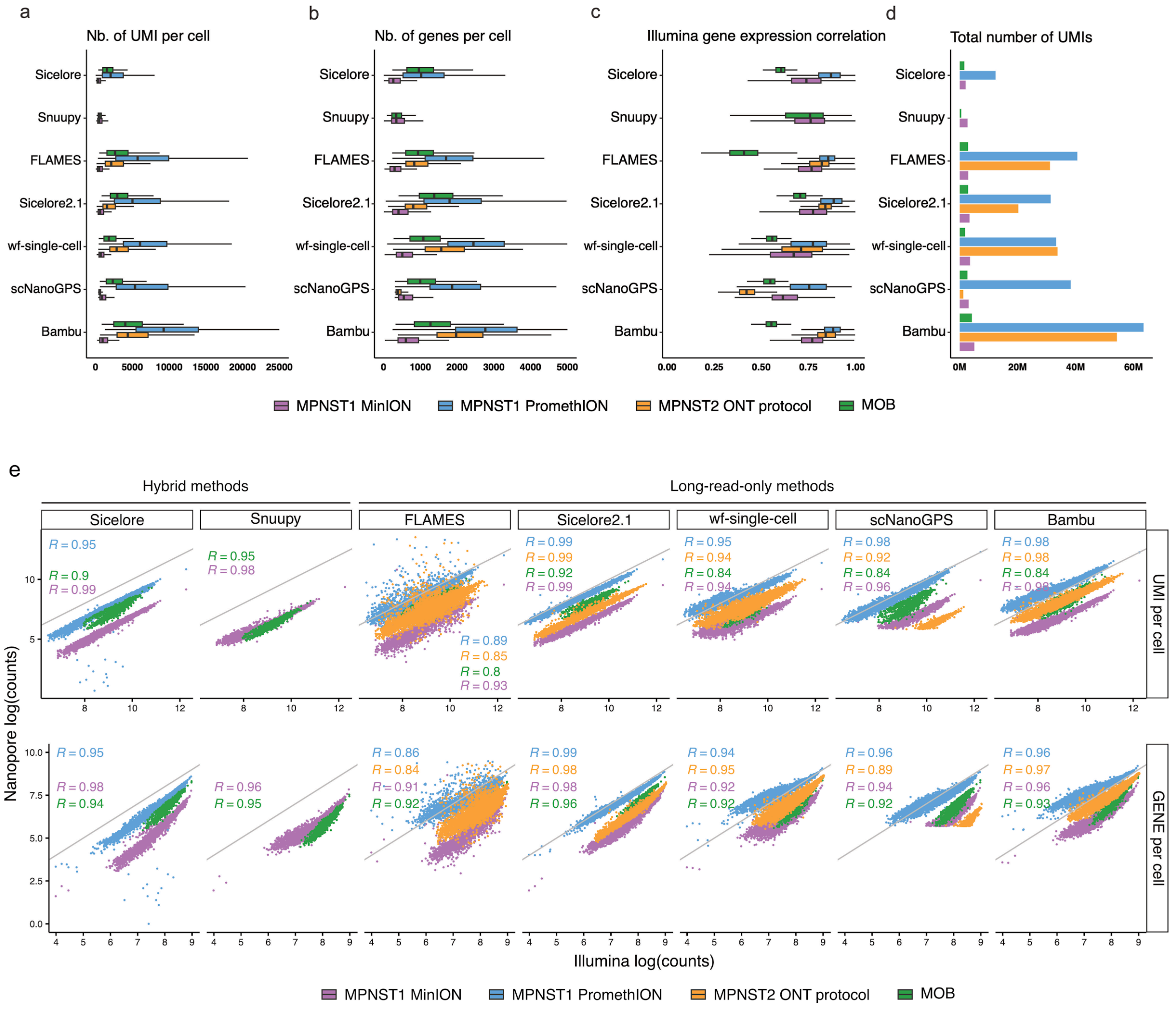

Figure S2 | Comparison of gene count matrices obtained from long-read versus short-read data.

The gene count matrices were obtained by processing the four Nanopore long-read datasets (see legend with associated colors) with each bioinformatics method listed in the y axis (panels a-d). Isosceles and IsoQuant are not included as they do not process reads into count matrices. **(a)** Number of UMIs per barcode. **(b)** Number of genes per barcode. **(c)** Box plot showing the distribution of Pearson correlation coefficient (r) of the number of UMI per gene between short and long read data. r values are calculated for each barcode, boxes represent the 25% quantile to 75% quantile range and the median. **(d)** Total number of detected UMIs per method. For runtime reasons, the MPNST2 dataset prepared with the sc-ONT standard protocol (in orange) was solely processed with the five long-read-only methods. **(e)** Scatter plots showing the number of UMI and number of genes per barcode (log-normalized values) detected in short read (x-axis) and long read (y-axis) data. The Pearson correlation coefficient for each dataset is shown in the same color. Gray lines represent the x=y relationship.

Distributed under a Creative Commons Attribution | CC-BY 4.0 International license

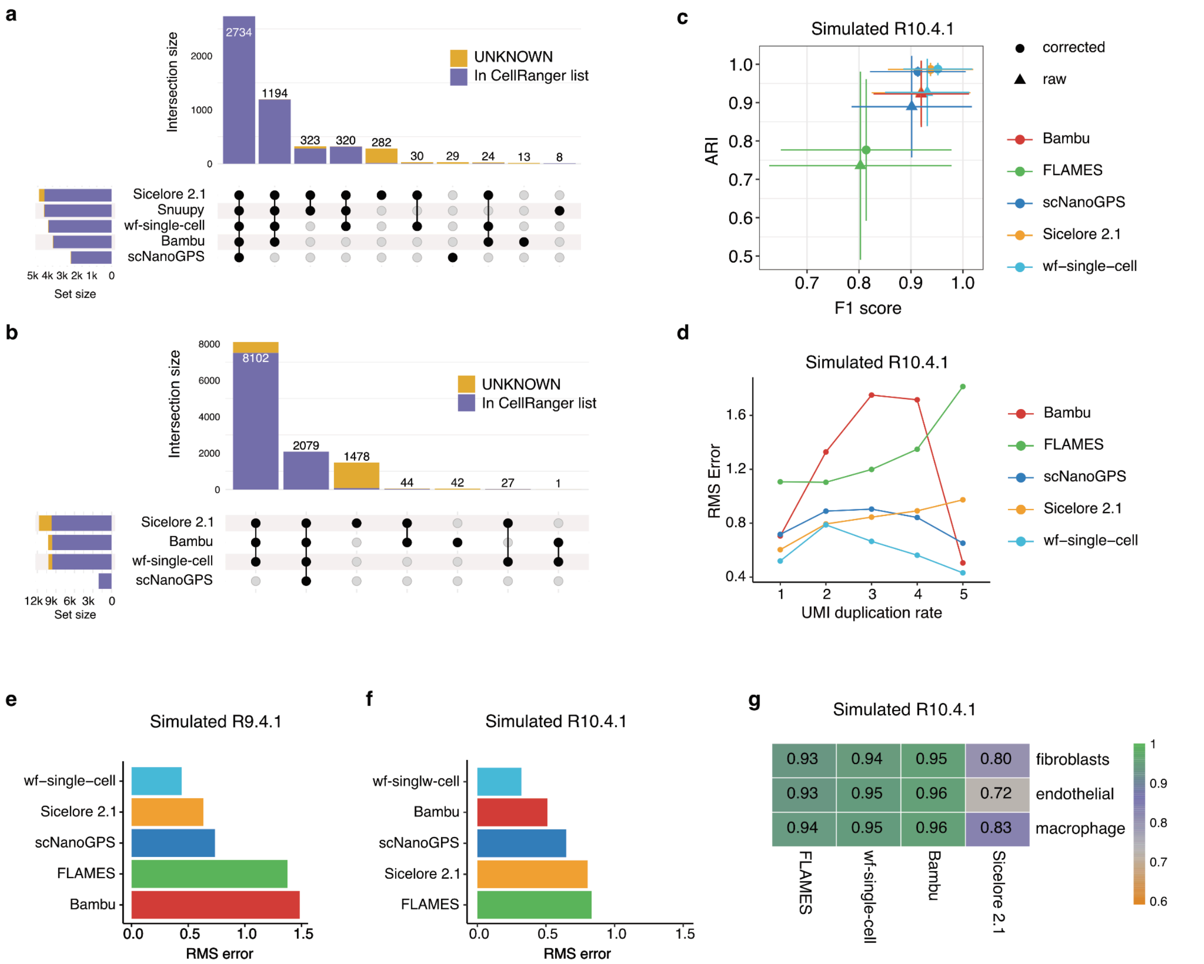

Figure S3 | Accuracy in barcode assignment and gene quantification fidelity.

(a-b) Barcode upset plot comparing different cell-associated barcode lists. The bar chart on the left shows the total number of barcodes found by each tool. The bar chart on top shows the number of barcodes in the intersection of shortlists from specific combinations of methods. The dots and lines underneath show the combinations. Plots are associated with MPNST1 scNaUmi-seq (MinION) (a) or MPNST2 ONT (PromethION) (b) datasets**.** **(c)** Scatter plots of the mean F1 score against mean ARI score for the UMI error correction. Error bars indicate the mean and standard error across simulated R10.4.1 datasets, on which methods run (n=2; UMI duplication 1 and 5). Bambu does not perform UMI error correction, results are shown based on raw UMI only. **(d)** Comparison of gene expression estimation by different methods on the simulated R10.4.1 datasets (1 million reads each) across five different UMI duplication rates, evaluated by root of mean square error (RMSE). **(e-f)** RMSE between the observed and expected gene expression values. RMSE were computed following normalization, where each expression matrix was standardized using the mean and standard deviation of the ground truth in the Simulated R9.4.1 **(e)** or R10.4.1 **(f)** data. **(g)** Pearson's correlation (color scale) of each method's isoform quantifications with expected quantification at pseudo-bulk level in R10.4.1 data.

Distributed under a Creative Commons Attribution | CC-BY 4.0 International license

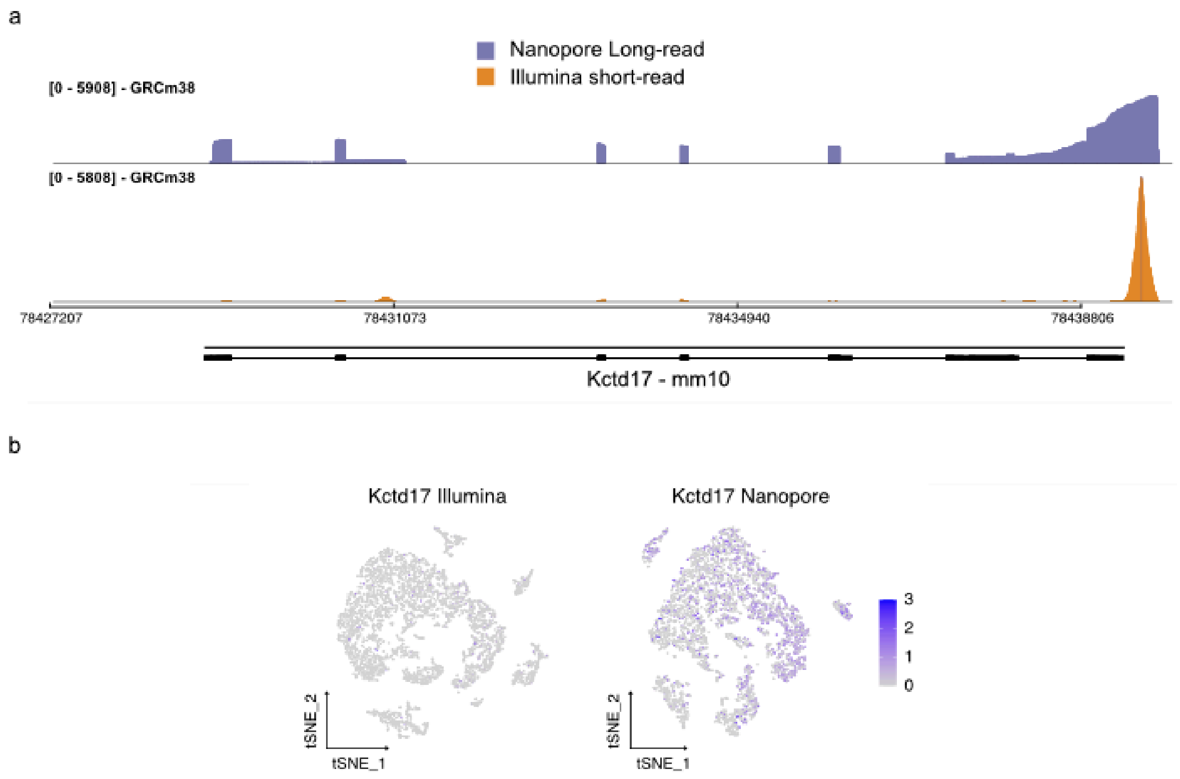

Figure S4 | Importance of gene annotation for isoform detection.

(a) Sashimi plot showing Illumina short reads and Nanopore long reads aligned to GRCm38 (mm10) mouse genome. Due to incomplete 3′ UTR annotation of *Kctd17* in ensemble annotation v86, a large fraction of short reads map to non-genic regions, leading to quantification errors in short-read data. **(b)** FeaturePlot of *Kctd17* gene in MPNST1 Illumina and PromethION data.

Distributed under a Creative Commons Attribution | CC-BY 4.0 International license

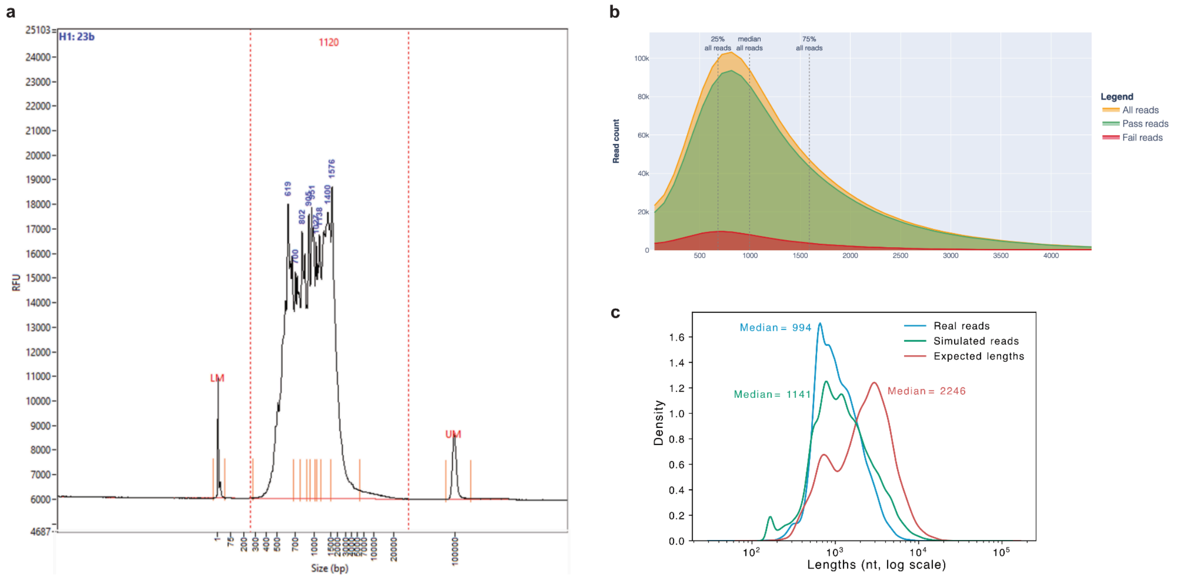

Figure S5 | Read length distributions of ScNaUmi-Seq PromethION data for MPNST1

(a) cDNA profile obtained after migration on the Agilent Fragment Analyzer (median fragment length: 1,120 bp). **(b)** Read length distribution after sequencing, generated with ToulligQC from the sequencing summary files. The plot shows all reads together with the subsets of passed and failed reads; vertical dashed lines indicate the 25th percentile, median (1,120bp), and 75th percentile of the read length distribution. The predominance of reads below 1 kb is consistent with previous reports for 10x Genomics–based single-cell long-read datasets, which typically show mean read lengths around ~850 bp (2). and a majority of reads shorter than 1 kb (3). **(c)** Read length distributions of real MPNST1 PromethION reads and simulated reads, together with the expected lengths of expressed transcripts in the MPNST1 dataset. The expected transcript length distribution was obtained by selecting expressed transcripts (UMI count > 1) from the wf-single-cell count matrix and retrieving their transcript lengths from the GENCODE M35 reference transcriptome.

Distributed under a Creative Commons Attribution | CC-BY 4.0 International license

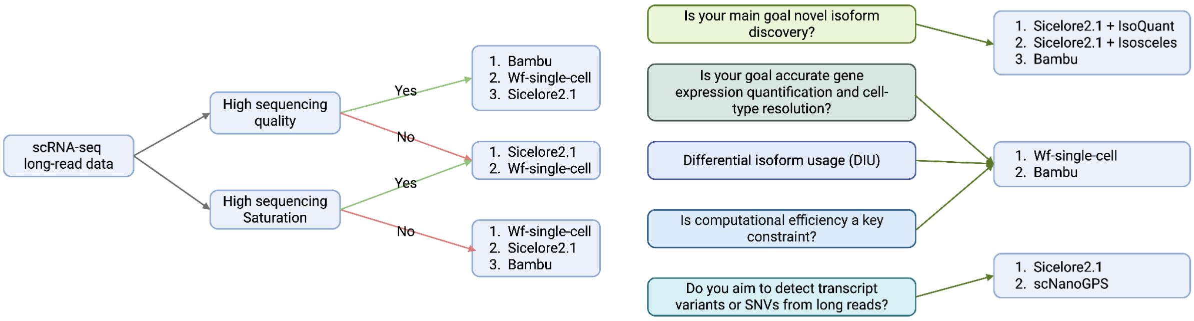

Figure S6 | Decision-tree guidelines for scRNA-seq long-read data analysis.

Distributed under a Creative Commons Attribution | CC-BY 4.0 International license

Supplementary Tables

Sample	Sequencing platform	Sequencing protocol	Sequencing kit	cDNA average size	Mean size pass	Sequencing saturation %	No. cells/spots	Total reads	Median phred score
MPNST1	ONT PromethION	scNaUmi-seq	SQK-LSK110	1338 bp	1319 bp	35%	4,559	151,334,327	11.32
MPNST1	ONT MinION	scNaUmi-seq	SQK-LSK109	1338 bp	1243 bp	4%	4,559	9,281,420	11.75
MPNST1	Illumina NextSeq 500	10x Genomics	-	-	-	81%	4,559	491,161,783	-
MPNST2	ONT PromethION	scNaUmi-seq	SQK-LSK110	1379 bp	980 bp	8%	10,297	130,507,091	14.0
MPNST2	ONT PromethION	ONT	SQK-PCS111	1341 bp	1000 bp	6.7%	10,297	108,009,263	13.56
MPNST2	Illumina NextSeq 500	10x Genomics	-	-	-	34%	10,297	401,224,345	-
MOB (Lebrigand et al 2023)	Illumina NextSeq 500	10x Genomics	-	-	-	93.1%	900	253,954,360	-
MOB (Lebrigand et al 2023)	ONT PromethION	scNaUmi-seq	SQK-LSK109	-	-	57%	900	10,226,285	11.81
Simulated R9.4.1	simulation	-	-	-	-	79%	635	20,000,707	13
Simulated R10.4.1	simulation	-	-	-	-	86%	635	20,000,261	20
Simulated dup1 R9.4.1	simulation	-	-	-	-	20%	4,559	990,690	13
Simulated dup2 R9.4.1	simulation	-	-	-	-	35%	4,559	1,006,813	13
Simulated dup3 R9.4.1	simulation	-	-	-	-	45%	4,559	991,685	13
Simulated dup4 R9.4.1	simulation	-	-	-	-	54%	4,559	995,366	13
Simulated dup5 R9.4.1	simulation	-	-	-	-	60%	4,559	991,390	13
Simulated dup1 R10.4.1	simulation	-	-	-	-	20%	4,559	990,690	20
Simulated dup2 R10.4.1	simulation	-	-	-	-	35%	4,559	1,006,813	20
Simulated dup3 R10.4.1	simulation	-	-	-	-	45%	4,559	991,685	20
Simulated dup4 R10.4.1	simulation	-	-	-	-	54%	4,559	995,366	20
Simulated dup5 R10.4.1	simulation	-	-	-	-	60%	4,559	991,390	20

Table S1 | Datasets used in this study.

MPNST1 and MPNST2-related data are accessible on ArrayExpress (accession number: E-MTAB-15190). MOB data are accessible on Gene Expression Omnibus (accession number: GSE153859). Simulated data are shared through Zenodo (record ID: 17312868)

Distributed under a Creative Commons Attribution | CC-BY 4.0 International license

Tool name	Programming language	Barcode demultiplexing	chimeric reads filtering	UMI error correction	UMI deduplication	Isoform analysis	Transcrip t discovery
Sicelore[1]	Java	short-read data	yes	SR data	consensus sequence	yes	—
Snuupy[2]	Python	short-read data	yes	SR data	consensus sequence	PolyA-based	—
scNapBar[3]	C++/Python/Perl	short-read data	—	SR data	—	yes	—
FLAMES[4]-BLAZE[5]	C++/Python	—	—	Merge UMIs within a fixed edit distance	keep longest read in each UMI group	yes	yes
wf-single-cell[6]	Python	10X inclusion list (count over threshold)	yes	UMI-tools clustering algorithm[11]	UMI-tools[11] representative	StringTie2 [12] FLAMES	yes
Sicelore 2.1[1]	Java	10X inclusion list	yes	Cluster UMIs based on edit distance	consensus sequence	yes	—
scNanoGPS[7]	Python	no guidance (count over threshold)	yes	Merge UMIs within a fixed edit distance	consensus sequence	LIQA[13]	—
Bambu[8]	R	10X inclusion list (count over threshold)	yes	—	keep longest read in each UMI group	EM algorithm	yes
Isosceles[9]	R	—	—	—	—	EM algorithm	yes
IsoQuant[10]	Python	—	—	—	—	EM algorithm	yes

Table S2 | Ten scRNA-seq long-read methods evaluated in this study.

*: paired dataset sequenced in short-read is used by hybrid approaches. BLAZE and FLAMES work together. References: 1 (Lebrigand et al. 2020), 2 (Long et al. 2021), 3 (Wang et al. 2021), 4 (You et al. 2023), 5 (Tian et al. 2021), 6 (https://github.com/epi2me-labs/wf-single-cell), 7 (Shiau et al. 2023), 8 (Sim et al. 2025), 9 (Kabza et al. 2024), 10 (Prjibelski et al. 2023), 11 (Smith et al. 2017), 12 (Kovaka et al. 2019) and 13 (Hu et al. 2021).

Distributed under a Creative Commons Attribution | CC-BY 4.0 International license

Tool	Version	Environment control	Link
Sicelore	v2.0	Docker	https://hub.docker.com/layers/hamraouii/sicelore/2.1/images/sha256-efbb9d562da211fb7806aa7ae70585652aee52aeca3a07526100a8b540e45f96
Snuupy	v0.2	Docker	https://hub.docker.com/layers/hamraouii/snuupy/2.0/images/sha256-4f931bbe6b4d436be35f861927d814de1d995ce2137dad5f31f35a58ec630146
scNapBar	v1.1.0	Conda	https://github.com/dieterich-lab/single-cell-nanopore/blob/master/environment.yaml
FLAMES	v0.1	Conda	https://github.com/LuyiTian/FLAMES
BLAZE	v1.1.0	Conda	https://github.com/shimlab/BLAZE/blob/main/conda_env/environment.yml
wf-single-cell	v3.0.3	Docker	https://hub.docker.com/layers/ontresearch/wf-single-cell/shab5b0dea0efc4685f74c8b4f91c979c587e23a020/images/sha256-1ef390dec17a6a1e2b0a51283d65cc57294e54e9bccb7d60a77bd677ffcc3926
Sicelore 2.1	v2.1	Docker	https://hub.docker.com/layers/hamraouii/sicelore/2.1/images/sha256-efbb9d562da211fb7806aa7ae70585652aee52aeca3a07526100a8b540e45f96
scNanoGPS	v1.1	Conda	https://github.com/gaolabtools/scNanoGPS/blob/v2.0/requirements.txt
Bambu	beta1.2	Singularity	https://hub.docker.com/layers/lingminhao/bambusc/beta1.2/images/sha256-74cfe91ff40a536a7f34175cfbd6cc34f9dd6ea18df4a8d9b31b869e0737e0cc
Isosceles	v0.2.0	Docker	https://hub.docker.com/layers/hamraouii/rstudio/1.1/images/sha256-9b0f1de5a694effe7a1653f91fcdaa2ad8740b57f38ebbb20c94b89f56988ae7
IsoQuant	v3.6.3	Docker	https://biocontainers.pro/tools/isoquant

Table S3 | Software version and environment control strategies

Distributed under a Creative Commons Attribution | CC-BY 4.0 International license

Supplementary Methods

Selected bioinformatics tools

The first key challenge in scRNA-seq Nanopore data analysis lies in the accurate identification of barcodes and UMI sequences. We selected the tools through an extensive and systematic review conducted between 2022 and 2025, aiming to be as comprehensive as possible. Tools not included were either outdated, relying on custom tooling or *ad hoc* workflows, or lacked sufficient documentation. In brief, the ten computational tools can be grouped in three categories: hybrid, long-read-only and isoform-specific methods (Figure 1b).

Hybrid methods, represented by Sicelore, Snuupy and scNapBar employ a hybrid sequencing approach in which barcodes and UMI assignment procedure is guided by short-read data. Sicelore extracts barcodes and UMI from short stretches of the long read between a valid adapter sequence and a threshold number of poly-A. Snuupy and scNapBar are developed as an improvement of Sicelore to either remove the dependency of the algorithm on the poly-A tail or the depth of short-read sequencing, respectively.

Conversely, long-read-only methods such as FLAMES, Sicelore 2.1, wf-single-cell, scNanoGPS, and Bambu, adopt long-read specific approaches, correcting barcodes and UMI without the use of paired short-read data. The long-read-only tools differ at three levels. Firstly, regarding barcode validation, FLAMES requires the cell-associated barcodes list as input, which can be generated by BLAZE or derived from a shortlist obtained from short-read sequencing data. Sicelore 2.1, wf-single-cell and Bambu (which relies on flexiplex) use an inclusion list (e.g. the ~3 million unique sequences for 10X Genomics v3 chemistry) and retain the most highly represented barcodes within a defined count threshold. scNanoGPS applies the iCARLO algorithm (4) to identify and correct erroneous barcodes, focusing particularly on the most abundant sequences. Secondly, tools differ by their assignment of UMI sequences to reads. FLAMES, Sicelore 2.1, and scNanoGPS cluster or merge UMIs mapped to the same gene or genomic interval using a Levenshtein distance threshold. In contrast, wf-single-cell employs the directional graph approach from UMI-tools (5) to perform UMI clustering, while Bambu directly merges raw UMI sequences. Finally, regarding the selection of representative reads within each UMI cluster, FLAMES and Bambu retain the longest read. wf-single-cell selects a representative read—typically the one with the highest read count or the most "central" UMI—using the default strategy of UMI-tools. In contrast, Sicelore 2.1 and scNanoGPS generate a consensus sequence across all reads in the cluster (Table S2).

Isoform-specific methods, such as IsoQuant and Isosceles, use existing preprocessing methods (e.g. Sicelore and wf-single-cell) for barcodes and UMI detection, and performs reference-guided *de novo* detection, quantification, and downstream analysis of isoforms at single-cell and pseudo-bulk levels.

Distributed under a Creative Commons Attribution | CC-BY 4.0 International license

Data

Single-cell cDNA library preparation and sequencing

Generation of single-cell cDNA

The MPNST1 and MPNST2 single-cell suspensions were converted into a barcoded scRNA-seq library with the 10x Genomics Chromium Single Cell 3′ Library, Gel Bead & Multiplex Kit and Chip Kit (v3), aiming for 20 00 cells following the manufacturer's instructions. The following modifications were applied to MPNST1: we extended the PCR elongation time during the initial PCR amplification of the cDNA from the manufacturer recommended 1 min to 3 min to minimize preferential amplification of small cDNAs (6).

Short-read Illumina libraries

Half of the amplified cDNA was used for short-read sequencing library preparation following the 10x Genomics protocol. MPNST1 was sequenced on an Illumina Nextseq 500 sequencer at GenomiqueENS facility, resulting in 491 M reads. MPNST2 was sequenced on an Illumina Nextseq 2000 sequencer at IMRB facility, resulting in 401 M reads.

Long-read ScNaUmi-seq protocol libraries

MPNST1 and MPNST2 libraries were prepared as described in Lebrigand et al (6), including the Optional steps for the depletion of cDNA lacking a terminal poly(A)/poly(T) tail. Nanopore sequencing libraries were prepared with the Oxford Nanopore SQK- LSK-110[MOU2] kit following the manufacturer's instructions. Sequencing was performed on R9.4.1 flow cells at GenomiqueENS facility (MPNST1 on MinION, MPNST2 on a P2solo) and at the Centre National de Recherche en Génomique Humaine (CNRGH) facility (MPNST1 PromethION).

Single-cell long-read ONT protocol libraries

The MPNST2 library was prepared with the "Single Cell sequencing on Promethion protocol" (https://nanoporetech.com/document/single-cell-transcriptomics-with-cdna-prepared-using-10x) with the SQK-PCS11 kit. We re-amplified 10 ng of the 10x Genomics PCR product for 4 cycles with 5′-CAGCTTTCTGTTGGTGCTGATATTGCAAGCAGTGGTATCAACGCAGAG-3′ and 5′ Biotine-CAGACACTTGCCTGTCGCTCTATCTTCCTACACGACGCTCTTCCGATCT 3′. After 0,8x AmpureXP purification to remove excess biotinylated primers, biotinylated is bound to Dynabeads™ M-280 Streptavidin beads (Invitrogen) and amplified with the primers cPRM for 4 cycles. Amplified cDNA was purified with 0,8x Ampure XP and sequenced on a R9.4.1 flowcell at GenomiqueENS facility on a P2solo according to the manufacturer's protocol.

Spatial scRNAseq long-read data

Distributed under a Creative Commons Attribution | CC-BY 4.0 International license

Spatial long-read data were downloaded from Gene Expression Omnibus (accession number: GSE153859), both as FASTQ and count matrix, according to the benchmarking steps. The subsequent processing was performed with the same pipeline as the non-spatial data.

Simulation of scRNAseq long-read data

To simulate scRNAseq Nanopore datasets (Table S1), the FASTQ file from the MPNST1 ScNaUmi-seq PromethION dataset was processed with wf-single-cell v3.0.3 using default parameters. The raw transcript count matrix was then analyzed using the Seurat package (v4.3.0). Normalization was performed using the NormalizeData function with the LogNormalize method, followed by log-transformation and scaling using ScaleData. A total of 2,000 variable features were selected, and 15 principal components were retained for dimensionality reduction. Clustering was performed using the Louvain algorithm with a resolution of 0.5, and cell type annotation was based on a predefined set of marker genes (detailed below). The transcript count matrix was then filtered to retain only macrophages, fibroblasts, and endothelial cells based on the annotation results. The filtered matrix was then used as input for AsaruSim v1.0.3 (7).

To generate realistic reads and replicate the error profile from the MPNST1 ScNaUmi-seq PromethION dataset (R9.4.1 Nanopore chemistry), a subset of the real FASTQ file was used as a reference model to simulate sequencing errors, and quality scores. The same subset of the real FASTQ file was aligned to the reference transcriptome using minimap2 (8) to derive an empirical truncation probability distributions on both 5′ and 3′ ends. A total of 39,816 transcripts were simulated across 635 cells. Subsequently, 20 million reads were randomly selected from the simulated pool following an 8-cycle PCR amplification. Additional FASTQ files were generated using the same procedure, but employing a real FASTQ file derived from R10.4.1 Nanopore chemistry as the reference model.

To simulate multiple datasets with various UMI duplication rates, we used the MPNST1 ScNaUmi-seq Illumina dataset as template to ensure a realistic read count distribution. We first identified 4,500 cell-associated barcodes using Cell Ranger v6.0.0 and used the corresponding UMI counts as input for AsaruSim v1.0.3 (--CB_counts option) to simulate long-read data. A total of 10,000 cDNA sequences were subsampled from the Ensembl release-113 mouse transcript reference with seqtk (https://github.com/lh3/seqtk) using a random seed value of 123 and used as reference transcriptome for AsaruSim. Five datasets were generated with increasing UMI duplication levels (--umi_duplication option) ranging from 1 to 5, and for both R9.4.1 and R10.4.1 Nanopore chemistries, using real datasets. Finally, 1 million reads were randomly selected from each simulated dataset, achieving sequencing saturation levels of 0.20, 0.35, 0.45, 0.54, and 0.60, respectively. After preprocessing the simulated datasets, we obtained raw UMI sequences and corrected UMI sequences resulting from a UMI error correction step. The corrected UMI were compared to the true UMI sequences (ground truth set in the simulation), measured with an F1-score.

Distributed under a Creative Commons Attribution | CC-BY 4.0 International license

Time and memory

To measure execution time and peak memory usage, reported in Figure S1, all jobs were executed on Linux servers equipped with Intel Xeon processors, 32 CPU cores, and 190 GB of memory. A maximum of 20 threads was allocated for all jobs. Execution time and maximum memory usage were assessed using the Linux time (*/usr/bin/time)* command with the *-v* flag. Execution time was extracted from the “Elapsed time” field, while peak memory consumption was recorded from the “Maximum resident set size” field.

Definitions

We call **barcode** the 16-nucleotide sequence used to uniquely identify droplets or spots. All the possible sequences are referenced in an **inclusion list** (formerly called whitelist), provided by 10X Genomics. After sequencing, barcodes are generally compared to the inclusion list to discard unreferenced sequences. The sequenced barcodes found in the inclusion list correspond to the barcode **shortlist**. This shortlist is then further filtered to remove empty droplets/spots. A knee plot, depicting the number of UMI associated with each barcode from the shortlist, enables distinguishing empty from non-empty droplets. The barcodes associated with non-empty droplets are finally called **cell-associated barcodes**.

Data processing

Tools and parameters associated with short-read data

For short-read (Illumina) MPNST datasets, BCL files were processed using 10x Genomics Cell Ranger software (v3.0.0). Reads were mapped onto a custom mouse transcriptome based on GRCm38 transcriptome, in which we added tdTomato sequence contained in the tumor cells. For Nanopore data Fast5 files, base calling was performed with Guppy v4.2.2 (https://nanoporetech.com/software/other/guppy), followed by read quality control using ToulligQC v2.3 (https://github.com/GenomiqueENS/toulligQC). The sequencing saturation curves were computed using CellRanger v6.0.0 for short-read data and wf-single-cell v3.0.1 for Nanopore data. The sequencing saturation values are given in Table S1.

Tools and parameters associated with long-read data

Snuupy and Sicelore were executed with recommended parameters. For both tools, barcode shortlisting was based on the CellRanger-provided inclusion list, using an edit distance of 2 for both barcode and UMI matching. Sicelore v2.1 nextflow workflow was also run independently with default parameters, without supplying an inclusion list.

Distributed under a Creative Commons Attribution | CC-BY 4.0 International license

FLAMES was executed by first running the precompiled C++ binary src/bin/match_cell_barcode with an edit distance of 2, a 12 nucleotide UMI length, and the CellRanger inclusion list (formerly whitelist) as input. Subsequently, the sc_long_pipeline.py script was executed with default parameters.

BLAZE was executed using --expected_cells set to 900 for spatial data, 4,500 for MPNST1, and 11,000 for MPNST2. The run included the following additional options: --high-sensitivity-mode, --emptydrop, and --out-CB-whitelist=whitelist_hs.

The wf-single-cell pipeline was executed with recommended parameters and the --expected_cells option set to 900 for spatial datasets, 4,500 for the MPNST1 dataset, and 11,000 for the MPNST2 dataset. In addition, the --barcode_max_ed parameter was set to 1 to improve barcode assignment accuracy.

scNanoGPS was run with default parameters, except for --CB_mrg_thr 1 and --CB_no_ext 0.3, which were adjusted to enhance barcode shortlist validation for PromethION and simulated datasets. For the simulated amplified datasets, the --min_gene_no parameter was set to 10 to prevent overly stringent filtering of gene count matrices.

For Isosceles, the tool was run using the recommended settings in two separate workflows: (1) with the deduplicated BAM file generated from step 4b (consensus sequences per UMI) of the Sicelore v2.1 Nextflow workflow, and (2) with the tagged BAM output of wf-single-cell. For the second workflow, the bam_to_tcc function was applied using the parameter barcode_tag = "CB". The tagged BAM files were deduplicated using the “dedup” command from UMI-tools (5). In both cases, the GTF annotation file provided by Isosceles contained only exonic features and was fixed to add transcripts using AGAT’s script agat_convert_sp_gxf2gxf.pl (9).

To run the long-read-only tools with spatial transcriptomics datasets, the barcode inclusion list visium-v1.txt was used in place of 3M-february-2018.txt, following guidance from 10x Genomics (https://kb.10xgenomics.com/hc/en-us/articles/115004506263-What-is-a-barcode-inclusion-list-formerly-barcode-whitelist).

Under these conditions, all tools received as input the FASTQ files, reference transcriptome sequence and annotation, and output a cells-by-isoforms count matrix. For some tools (detailed below), isoform discovery is performed and the output additionally includes the new annotation file.

All software environments and version details are provided in Table S3.

Accuracy in barcodes and UMI assignment

Evaluation of barcode false-positive detection

To assess the false-positive detection rate in barcode calling for each method in Figure 2b, we focused on tools that identify cell-associated barcodes based on read count thresholds: BLAZE, wf-single-cell, scNanoGPS, and Bambu. These methods detect barcodes by counting their

Distributed under a Creative Commons Attribution | CC-BY 4.0 International license

occurrences and selecting the most abundant ones as cell-associated (shortlisted) barcodes, typically using a threshold determined by the inflection point in the knee plot of barcode rank versus UMI count. To evaluate performance, we computed precision-recall curves across varying count thresholds, using the shortlist of barcodes identified by CellRanger on short-read data (raw count matrix) as the ground truth. Prior to this, empty droplets were filtered out using the DropletUtils R package (10), with default parameters and a false discovery rate (FDR) threshold of 1%. Barcodes identified in long-read data but not present in the CellRanger-filtered shortlist were considered false positives, while true positives were defined as barcodes present in both sets.

Accuracy of UMI error correction

To generate Figure 2c-d, for each tool, UMI sequences were extracted from tagged BAM files prior to the deduplication step using bioalcidaejdk (11). Raw and corrected UMI sequences were obtained as follows: for Sicelore 2.1, the 'U7' tag was used for raw UMIs and the 'U8' tag for corrected UMIs; for wf-single-cell, the 'UR' and 'UB' tags denoted raw and corrected UMIs, respectively. In the case of FLAMES, raw UMIs were parsed from read names in the realign2transcript.bam file, while corrected UMIs were extracted using a custom script following the UMI merging step. For scNanoGPS, raw UMI sequences were retrieved from the barcode_list.tsv file, and corrected UMIs were extracted from read names after the 'curation' step. For Bambu, raw UMIs were obtained from the demultiplexed.bam file.

To evaluate UMI correction accuracy, we compared the corrected and raw UMIs against the ground-truth UMI sequence from simulated data. Precision, recall, and F1-score were calculated using the following definitions. A true positive (TP) was defined as a corrected UMI that matched the ground-truth UMI. Precision was computed as the proportion of correctly corrected UMIs out of all corrected UMIs (TP / total corrected), while recall was the proportion of correctly corrected UMIs out of all ground-truth UMIs (TP / total true). The F1-score was computed as the harmonic mean of precision and recall:

$$F1\ score\ = 2\,.\,\frac{precision \times recall}{precision + recall}$$

In addition, clustering accuracy of UMI grouping was assessed using the Adjusted Rand Index (ARI), computed between the true UMI group labels and the predicted UMI clusters provided by each tool. All analyses were implemented in R using traceable R Markdown notebooks.

Count matrix processing

Projection and cell type annotation

The 10x Cell Ranger filtered matrix output files were imported into Seurat (v4.3.0) (12). For all samples and for quality control purposes, genes expressed in less than three cells were discarded, and cells with less than 300 unique gene counts were filtered out. The gene expression matrix was

Distributed under a Creative Commons Attribution | CC-BY 4.0 International license

normalized with Seurat's LogNormalize function and 2000 variable features were selected using Seurat's vst method then the matrix was scaled using Seurat's linear model. A PCA was generated using Seurat's RunPCA function. Then, cells were clustered using the Louvain algorithm, with 15 principal components as input of Seurat's FindNeighbors function and a resolution of 0.5 for Seurat's FindClusters function. Single cells were annotated for cell type using a modified version of Seurat's AddModuleScore function and cell type-specific marker gene sets (see Code and Data availability).

Pseudo-bulk expression correlation analysis

Pseudo-bulk analyses were used for Figure 2f, Figure S3g and Figure 5b. To compare pseudo-bulk expression profiles between methods, Seurat objects were annotated for cell types. Gene counts were aggregated across cells within each cell type to generate pseudo-bulk matrices. These matrices were normalized using counts-per-million followed by log-transformation. Pearson correlation coefficients were then computed between matched cell types across methods.

Quantitative assessment of cell type annotation and projection

To evaluate the biological resolution achieved by each method, the quality of cell type annotation and projection was assessed (Figure 3 and Figure 4).

First, individual cells were annotated based on specific gene markers, following the annotation protocol described above. We computed the Local Inverse Simpson's Index (LISI) (13), a diversity score designed to assess both batch mixing (iLISI) and cell-type separation (cLISI). The cLISI score represents the effective number of cell type labels present in the neighborhood of each cell on the UMAP projection. cLISI scores were computed for each method using the compute_lisi function from the lisi R package, applied to the UMAP reduction of cell type–annotated Seurat objects.

Next, the long-read gene count matrix was integrated with the short-read count matrix using Seurat's CCAIntegration method, after which cLISI scores were computed again using the same compute_lisi function. To assess the similarity between cell type labels derived from short-read and long-read data, we used standard clustering evaluation metrics, including the Adjusted Rand Index (ARI).

For spatial transcriptomics datasets, LISI was adapted to quantify the effectiveness of spatial domain detection. In this context, the LISI score reflects the effective number of spatial domain labels present within the local neighborhood of each spot, using a fixed 5-spot window. In addition to LISI and ARI, we employed the CHAOS score to evaluate the spatial continuity of the detected domains (14, 15). The CHAOS score quantifies intra-domain spatial compactness by calculating the average distance to the nearest neighbor within each domain, normalized by the total number of spots. Lower CHAOS values indicate more spatially coherent clusters.

Distributed under a Creative Commons Attribution | CC-BY 4.0 International license

Differential isoform analysis

Differential isoform analysis was performed using the Isoswitch R package (https://github.com/ucagenomix/isoswitch). Results are presented in Figure 5c and Figure 6. For each tool, gene- and transcript-level count matrices were used to construct a multi-assay Seurat object. As ground truth, we used the true gene- and isoform-level count matrices generated by the AsaruSim simulation framework. A ground-truth multi-assay Seurat object was built from these simulated matrices. Each assay was normalized independently using the LogNormalize method. Clustering was performed based on the gene-level assay. Cell-type annotation was established on the ground-truth object based on cell type-specific marker gene scores (see the “Count matrix processing” section of the Supplementary Methods). This ground-truth cell-type annotation was then transferred to the method-specific multi-assay objects, so that differential isoform usage was assessed across the same cell groups for all methods. The set of 18 DSGs identified from the simulated ground-truth object using the same statistical thresholds ($p < 0.05$ and absolute log2FC > 2) was used as the reference against which method-specific DSG predictions were compared.

Differential isoform usage was then assessed using the ISO_SWITCH_ALL function from Isoswitch, which leverages Seurat’s FindMarkers function to identify differentially spliced genes (DSGs) for each cell type (macrophages, fibroblasts, and endothelial cells). The resulting lists of DSGs were used to generate an UpSet plot to visualize overlaps across tools (Figure 5d and Figure 6a). In addition, log fold changes (logFC) were computed and correlated with those from the ground truth to assess consistency in differential isoform detection.

Gene ontology analysis

DSGs were filtered for p-value < 0.01 and absolute value of the average log2FC > 2. For gene ontology analysis, the list of 2942 significant DSGs was provided as input (gene names) to the clusterProfiler’s enrichGO function (version 4.6.2) using default parameters (16). Top 8 ontologies were visualized using enrichplot (version 1.18.4).

Genes and isoforms identification and quantification

We used the simulated data to assess the accuracy of transcript assignment.

Accuracy of reads assignment to isoforms

Transcript labels were extracted from tagged BAM files using bioalcidaejdk (11). The ‘TR’ tag was used for Sicelore 2.1, and the ‘IT’ tag for wf-single-cell. For FLAMES, transcript labels were parsed from the realign2transcript.bam file. For scNanoGPS, a customized version of LIQA (17) was used to extract read names and their assigned transcript labels. To evaluate transcript assignment accuracy, assigned transcript labels were compared to the simulated ground-truth transcript IDs. For each transcript class, precision and recall were computed using parallel processing across all simulation

Distributed under a Creative Commons Attribution | CC-BY 4.0 International license

cycles. The F1-score was then calculated as the harmonic mean of precision and recall. Precision, recall, and F1-score values were computed per tool for 1 and 5 UMI duplication simulation, and summarized as mean ± standard deviation for each metric. Results are shown on Figure 5a.

Comparison of gene and isoform expression estimation to ground truth

To assess the accuracy of gene and isoform expression quantification in Figure 7b, we compared the output of each method to the simulated ground truth using the root of mean squared error (RMS error, RMSE). The formula behind the mean squared error is:

$$MSE = \frac{1}{n} \sum_{i=1}^{n} \left(x_i - y_i\right)^2$$

where x_i and y_i represent the estimated and true expression values for gene i , respectively.

To control for variation in sequencing depth and mitigate library size biases, each expression matrix (estimated and ground truth) was standardized using the mean and standard deviation derived from the ground truth. After normalization, the RMSE was calculated between the normalized matrices to provide a more robust comparison of expression accuracy at the single-cell level. All evaluations were performed separately for each simulation and method. Results are shown on Figure 2d and Figure S3d.

Novel isoform prediction

Isoform prediction, or transcriptome re-annotation, is a feature integrated in some tools (Bambu, IsoQuant, FLAMES, Isosceles and wf-single-cell). We evaluated the output annotation files as follows.

Discovery of novel isoforms in simulated data

To evaluate the accuracy of novel transcript prediction, we generated a reduced reference annotation by excluding 15% (4,412 transcripts) of the 29413 expressed transcripts from the GENCODE M35 annotation used in the simulated dataset. The excluded transcripts were treated as the true novel isoforms, while the remaining annotated transcripts were considered as true known isoforms.

The incomplete reference annotation, along with the full set of simulated reads, was then provided to isoform prediction tools: wf-single-cell, FLAMES, and Bambu. For Isosceles, raw reads were first processed using Sicelore v2.1 or wf-single-cell then tagged and deduplicated BAM files were used as input. The resulting transcriptome assemblies, output in GTF format, were then parsed to classify transcripts as either known or novel based on the annotation status in the GTF files.

To assess accuracy, we used gffcompare to compare the predicted novel transcripts against the set of true novel isoforms. Precision and sensitivity were computed, and the F1-score was calculated as the harmonic mean of precision and sensitivity. Results are shown on Figure 7d.

Distributed under a Creative Commons Attribution | CC-BY 4.0 International license

Consistency among predicted annotations on real data

To evaluate the overlapping between annotations, we used gffcompare to pairwise compare the full annotation which was predicted by each tool (predicted_annotations.txt lists all the annotation files) and the original annotation (ref_annotation.gff). We used the command gffcompare -g ref_annotation.gff -i predicted_annotations.txt. This command outputs isoform class codes that were converted to SQANTI classification (18). The results are visualized using an upset plot on Figure 5d.

Distributed under a Creative Commons Attribution | CC-BY 4.0 International license

References

1. Fu,Y., Kim,H., Roy,S., Huang,S., Adams,J.I., Grimes,S.M., Lau,B.T., Sathe,A., Ji,H.P. and Zhang,N.R. (2025) Single cell and spatial alternative splicing analysis with Nanopore long read sequencing. *Nat. Commun.*, **16**, 6654.

2. Scoones,A.L.A., Lan,Y., Utting,C., Pouncey,L., Lister,A., Kudasheva,S., Mehta,N., Irish,N., Swarbreck,D., Gharbi,K., *et al.* (2025) A comparison of long-read single-cell transcriptomic approaches. *bioRxiv*, 10.1101/2025.07.03.662955.

3. Hansen,M.S., Hill,C.J., Sussel,L. and Wells,K.L. (2025) Optimizing Single-Cell Long-Read Sequencing for Enhanced Isoform Detection in Pancreatic Islets. *bioRxiv*, 10.1101/2025.04.30.651101.

4. Shiau,C.-K., Lu,L., Kieser,R., Fukumura,K., Pan,T., Lin,H.-Y., Yang,J., Tong,E.L., Lee,G., Yan,Y., *et al.* (2023) High throughput single cell long-read sequencing analyses of same-cell genotypes and phenotypes in human tumors. *Nat. Commun.*, **14**, 4124.

5. Smith,T., Heger,A. and Sudbery,I. (2017) UMI-tools: modeling sequencing errors in Unique Molecular Identifiers to improve quantification accuracy. *Genome Res.*, **27**, 491–499.

6. Lebrigand,K., Magnone,V., Barbry,P. and Waldmann,R. (2020) High throughput error corrected Nanopore single cell transcriptome sequencing. *Nat. Commun.*, **11**, 4025.

7. Hamraoui,A., Jourdren,L. and Thomas-Chollier,M. (2025) AsaruSim: a single-cell and spatial RNA-Seq Nanopore long-reads simulation workflow. *Bioinformatics*, 10.1093/bioinformatics/btaf087.

8. Li,H. (2018) Minimap2: pairwise alignment for nucleotide sequences. *Bioinformatics*, **34**, 3094–3100.

9. Dainat,J., Hereñú,D. and Pucholt,P. (2020) AGAT: Another Gff Analysis Toolkit to handle annotations in any GTF. *GFF Format*, **10**.

10. participants in the 1st Human Cell Atlas Jamboree, Lun,A.T.L., Riesenfeld,S., Andrews,T., Dao,T.P., Gomes,T. and Marioni,J.C. (2019) EmptyDrops: distinguishing cells from empty droplets in droplet-based single-cell RNA sequencing data. *Genome Biol.*, **20**, 63.

11. Lindenbaum,P. and Redon,R. (2018) bioalcidae, samjs and vcffilterjs: object-oriented formatters and filters for bioinformatics files. *Bioinformatics*, **34**, 1224–1225.

12. Hao,Y., Hao,S., Andersen-Nissen,E., Mauck,W.M., Zheng,S., Butler,A., Lee,M.J., Wilk,A.J., Darby,C., Zager,M., *et al.* (2021) Integrated analysis of multimodal single-cell data. *Cell*, **184**, 3573-3587.e29.

13. Korsunsky,I., Millard,N., Fan,J., Slowikowski,K., Zhang,F., Wei,K., Baglaenko,Y., Brenner,M., Loh,P. and Raychaudhuri,S. (2019) Fast, sensitive and accurate integration of single-cell data with Harmony. *Nat. Methods*, **16**, 1289–1296.

Distributed under a Creative Commons Attribution | CC-BY 4.0 International license

14. Guo,L., Hu,Z., Zhao,C., Xu,X., Wang,S., Xu,J., Dong,J. and Cai,Z. (2021) Data Filtering and Its Prioritization in Pipelines for Spatial Segmentation of Mass Spectrometry Imaging. *Anal. Chem.*, 10.1021/acs.analchem.0c05242.

15. Alexandrov,T. and Bartels,A. (2013) Testing for presence of known and unknown molecules in imaging mass spectrometry. *Bioinformatics*, **29**, 2335–2342.

16. Yu,G., Wang,L.-G., Han,Y. and He,Q.-Y. (2012) clusterProfiler: an R package for comparing biological themes among gene clusters. OMICS, 16, 284–287. https://doi.org/10.1089/omi.2011.0118

17. Hu,Y., Fang,L., Chen,X., Zhong,J.F., Li,M. and Wang,K. (2021) LIQA: long-read isoform quantification and analysis. *Genome Biol.*, **22**, 1–21.

18. Tardaguila,M., Fuente,L. de la, Marti,C., Pereira,C., Pardo-Palacios,F.J., Risco,H. del, Ferrell,M., Mellado,M., Macchietto,M., Verheggen,K., *et al.* (2018) SQANTI: extensive characterization of long-read transcript sequences for quality control in full-length transcriptome identification and quantification. *Genome Res.*, **28**, 396–411.

Distributed under a Creative Commons Attribution | CC-BY 4.0 International license
